# Machine Learning-Assisted FTIR Spectroscopy Analysis of Kidney Preservation Fluids for Delayed Graft Function Risk Stratification

**DOI:** 10.3390/jcm15072762

**Published:** 2026-04-06

**Authors:** Luis Ramalhete, Rúben Araújo, Miguel Bigotte Vieira, Emanuel Vigia, Ana Pena, Sofia Carrelha, Cristiana Teixeira, Anibal Ferreira, Cecilia R. C. Calado

**Affiliations:** 1 Blood and Transplantation Center of Lisbon, Instituto Português do Sangue e da Transplantação, Alameda das Linhas de Torres, n◦ 117, 1769-001 Lisbon, Portugal; 2 NOVA Medical School, Faculdade de Ciências Médicas, Universidade NOVA de Lisboa, 1169-056 Lisbon, Portugal; 3 iNOVA4Health—Advancing Precision Medicine, Núcleo de Investigação em Doenças Renais, NOVA Medical School, Faculdade de Ciências Médicas, Universidade NOVA de Lisboa, 1169-056 Lisbon, Portugal; 4 LBS—Lisbon Business & Government School, Rua de São Bernardo 34-A, 1200-427 Lisbon, Portugal; 5 Nephrology, Hospital Curry Cabral, Unidade Local de Saúde de São José, R. da Beneficência 8, 1050-099 Lisbon, Portugal; 6 Hepatobiliopancreatic and Transplantation Center, Curry Cabral Hospital, Unidade Local de Saúde de São José, R. da Beneficência 8, 1050-099 Lisbon, Portugal; 7 Centro Clínico Académico de Lisboa, Faculdade de Ciências Médicas, Universidade NOVA de Lisboa, 1169-056 Lisbon, Portugal; 8 ISEL—Instituto Superior de Engenharia de Lisboa, Instituto Politécnico de Lisboa, Rua Conselheiro Emídio Navarro 1, 1959-007 Lisbon, Portugal; 9 Institute for Bioengineering and Biosciences (iBB), The Associate Laboratory Institute for Health and Bioeconomy-i4HB, Instituto Superior Técnico (IST), Universidade de Lisboa (UL), Av. Rovisco Pais, 1049-001 Lisbon, Portugal

**Keywords:** kidney transplantation, delayed graft function, preservation fluid, FTIR spectroscopy, fingerprint region, machine learning, donor-blinded validation, calibration, decision curve analysis

## Abstract

**Background/Objectives**: Delayed graft function (DGF) remains a common early complication after deceased donor kidney transplantation and is challenging to anticipate using routine pre-implant clinical variables alone. We investigated whether high-throughput Fourier transform infrared (FTIR) spectroscopy of static cold storage preservation fluid (not machine perfusion perfusate) captures biochemical information associated with DGF and warrants further evaluation alongside routine pre-implant clinical predictors. **Methods**: In this single-center retrospective cohort, we analyzed preservation fluid samples from 56 kidney transplants originating from 49 deceased donors (7 donors contributed two kidneys); DGF occurred in 14/56 (25.0%). Dried-film FTIR spectra were acquired using a plate-based high-throughput accessory, and analyses focused on the fingerprint region (900–1800 cm^−1^) with prespecified preprocessing and quality control. We developed and compared clinical-only, FTIR-only, and combined predictive models and estimated performance using donor-blinded 5-fold StratifiedGroupKFold cross-validation (grouped by donor code) to prevent leakage across paired kidneys. **Results**: Donor-blinded discrimination (pooled out-of-fold ROC-AUC) was 0.775 for the clinical-only model, 0.814 for the FTIR-only model, and 0.796 for the combined model; probabilistic accuracy (Brier score; lower is better) was 0.162, 0.194, and 0.177, respectively. Calibration intercepts were negative and slopes were <1, indicating overly extreme risk estimates under strict donor-blinded validation and supporting recalibration prior to deployment. Decision curve analysis suggested a positive net benefit for clinically plausible thresholds. **Conclusions**: These findings support the feasibility of rapid, low-cost FTIR profiling of routinely available preservation fluid as a proof-of-concept approach for exploratory DGF risk stratification, rather than as a clinically deployable prediction tool. Given the small sample size and the instability of subgroup estimates, the main next steps are external validation in larger multicenter cohorts, prospective workflow studies, and model updating/recalibration.

## 1. Introduction

Kidney transplantation (KT) remains the most effective long-term therapy for end-stage kidney disease, but the first days after implantation are still a high-risk interval in which early allograft dysfunction can set the trajectory for months or years. One reason this problem has not softened with time is that modern transplant programs increasingly depend on higher-risk deceased donor kidneys, particularly from donation after circulatory death (DCD), alongside the more traditional donation after brain death (DBD), to narrow the gap between supply and demand. DCD expansion has saved lives, yet it also raises the biological “injury load” at baseline because DCD grafts are exposed to warm ischemia around circulatory arrest, which compounds cold ischemia and amplifies ischemia–reperfusion injury (IRI) at reperfusion [1,2]. In parallel, even in DBD transplantation, the hemodynamic and inflammatory consequences of brain death can prime the kidney for endothelial activation and tubular stress, meaning DBD and DCD are not merely administrative categories as they often represent different starting points for the same downstream pathophysiology [1].

In daily clinical practice, delayed graft function (DGF) remains the most visible early manifestation of this injury. DGF is most commonly defined pragmatically as dialysis within the first 7 days after transplantation; this is the dominant definition used across much of the literature, but it is also an imperfect surrogate because dialysis initiation is shaped by local practice, recipient context, and clinician thresholds [1,2,3]. A systematic review highlighted striking heterogeneity in how DGF is defined and diagnosed across studies, which complicates comparisons between cohorts and can quietly undermine any model trained in one setting and deployed in another [1]. Even so, DGF is not a trivial early inconvenience. Large registry-based work has linked DGF to an increased risk of death with a functioning graft, emphasizing that early dysfunction can reflect a broader peri-transplant vulnerability state rather than a kidney-only delay [4]. Meta-analytic evidence supports associations between DGF and adverse outcomes such as graft failure and acute rejection, reinforcing the clinical logic for earlier risk stratification and tailored post-transplant management in patients at high DGF risk [5]. At the same time, emerging data claims that the binary DGF label hides clinically meaningful gradients: the duration of DGF carries prognostic information, with prolonged DGF, rather than DGF itself, showing the clearest relationship with inferior graft survival, a nuance that matters when one tries to translate prediction into action [6,7].

Biologically, DGF can be framed as the clinical endpoint of IRI-driven acute kidney injury in the allograft. IRI is not simply a transient “stunning” phenomenon; it involves coordinated injury and repair programs including mitochondrial stress, oxidative injury, microvascular dysfunction, and innate immune activation that can prolong functional recovery [8]. Crucially, IRI also intersects with alloimmunity. In contemporary kidney transplant cohorts, DGF has been associated with a higher risk of biopsy-proven acute rejection, suggesting that the injury environment may still amplify alloimmune responses even under modern immunosuppression [9,10,11]. Earlier clinical work already hinted that DGF and rejection interact in shaping outcomes, reinforcing the idea that early injury phenotyping may influence not just dialysis planning, but surveillance intensity and the threshold for biopsy in a period where ischemic dysfunction and alloimmune injury can overlap clinically [12]. This link becomes even more relevant as transplant immunology moves toward more nuanced risk models that integrate both injury biology and recipient immune readiness, particularly in settings where donor-specific antibodies or other immune risks are present [13].

Preservation and logistics remain decisive contributors to early injury. Cold ischemia time (CIT) is a robust risk factor, and large observational work has shown that each additional hour of CIT increases the risk of graft failure and mortality after transplantation—an effect that keeps preservation strategy firmly in the causal chain of early outcomes [14]. These realities help explain why machine perfusion has shifted from a technical niche toward a clinically meaningful tool in higher-risk transplantation. A landmark randomized trial demonstrated that hypothermic machine perfusion reduced the risk of DGF compared with static cold storage and improved early graft outcomes [15]. Preservation strategies have continued to evolve, including oxygenated hypothermic perfusion in DCD kidneys (tested in a paired, randomized phase 3 trial) and normothermic machine perfusion approaches designed to recondition or assess the organ closer to implantation under near-physiologic conditions [16,17].

Beyond their therapeutic promise, these platforms create a translational opportunity: they generate perfusion fluids that can serve as a practical, noninvasive “liquid biopsy” of the organ during the preservation window, when decisions are still actionable. Perfusate sampling is scalable and workflow-friendly compared with pre-implant biopsies, but the biomarker literature has faced a recurring limitation: single analytes often show associations with early function in one cohort yet struggle with reproducibility, incremental value over clinical variables, or transfer across centers and platforms [18]. A systematic review focusing on perfusate biomarkers produced during hypothermic machine perfusion reached a similar conclusion: many candidates are biologically interesting, but no single marker appears sufficient across settings, supporting the move toward multi-signal strategies and rigorous validation [18]. More broadly, the growing clinical interest in liquid biopsy strategies across urologic diseases reinforces the translational value of noninvasive biofluid-based profiling platforms [19,20].

Metabolomics strengthens this argument by showing that perfusate contains structured biological information linked to organ injury and outcomes. In Kidney International, untargeted metabolomics of perfusate collected during hypothermic machine perfusion identified metabolic signatures associated with perfusion context and allograft failure, reinforcing that perfusate chemistry reflects meaningful organ biology rather than random noise [21,22]. Yet omics pipelines can be costly and operationally difficult to deliver in real time at the point of transplant, which is where rapid, information-dense measurements become attractive.

Fourier transform infrared (FTIR) spectroscopy fits this niche because it is fast, label-free, and relatively low cost per sample once established, capturing composite biochemical structure (proteins, lipids, carbohydrates, metabolites) in a single spectrum. However, the clinical translation of FTIR spectral data depends strongly on rigorous quality control, including standardized sample handling, instrument-aware acquisition, and reproducible spectral preprocessing, particularly when analyzing biofluids and water-dominant matrices [23]. Our group has shown that FTIR spectral fingerprints in pre-biopsy serum carry clinically actionable immune injury information, enabling prediction of cellular (T cell-mediated) rejection and, in a subsequent cohort, discrimination between T cell-mediated and antibody-mediated rejection [24,25]. In a recent pilot work, rapid FTIR spectral fingerprinting of kidney allograft perfusion fluids distinguished DCD from DBD donors using a machine learning framework, supporting feasibility and suggesting that donor pathway biology is detectable in perfusate spectra [22]. Building on that pilot work, the present study evaluates whether FTIR profiling of static cold storage preservation fluid can predict the clinical endpoint of DGF.

In this study, two donor pathways (DBD and DCD) and two preservation/perfusion solutions (Celsior^®^ (Genzyme, Cambridge, MA, USA) and Custodiol^®^/HTK (Pharma, Newtown, PA, USA)) were included. Although these solutions show broadly comparable clinical performance on average across trials and meta-analyses, they differ in composition and can impose distinct biochemical and spectroscopic “backgrounds” on the perfusate. If not handled explicitly, these solution-specific signatures may act as hidden confounders in spectral modeling and compromise downstream inference [26]. Comparative work also underscores that solution choice and preservation details can influence DGF incidence in certain contexts, so solution type should be treated as a structured source of variation rather than an incidental covariate [26,27,28,29]. The same applies to donor pathway: systematic reviews consistently report higher DGF risk in DCD kidney transplantation compared with DBD, even when longer-term outcomes can be acceptable, meaning that a model can appear accurate simply by learning “DCD-ness” unless the design forces it to learn injury signals that generalize beyond donor type [30].

Machine learning is well suited to FTIR spectroscopy because spectra are high-dimensional and intrinsically collinear. For clinical translation, it is also necessary to consider transparency, bias control, and evaluation in decision-relevant terms. Reporting standards have evolved accordingly: the TRIPOD statement remains foundational for prediction model studies, and the TRIPOD+AI guideline updates this framework for regression and machine-learning approaches, reflecting the field’s expectation that development, tuning, validation, and reporting be explicit and auditable [31,32]. In parallel, PROBAST provides structured assessment of risk of bias and applicability for prediction models, and PROBAST+AI extends this to modern regression and artificial intelligence (AI)/machine learning (ML) pipelines, emphasizing that dataset design, predictor handling, and validation choices can be just as important as the final performance metric [33,34]. Finally, if the goal is to influence care, model assessment should include clinical utility, not only discrimination. Decision curve analysis (DCA) was introduced to quantify net benefit across plausible threshold probabilities, allowing comparison of a model-based strategy to defaults such as “treat all” or “treat none,” and later work clarified how DCA should be reported and interpreted alongside discrimination and calibration [35,36,37].

## 2. Materials and Methods

### 2.1. Study Design and Cohort

#### 2.1.1. Study Design, Setting, and Timeframe

This was a single-center, observational study designed to evaluate whether FTIR spectral fingerprints obtained from kidney preservation fluid samples that had been collected during routine static cold storage can predict DGF after deceased donor kidney transplantation. The study was conducted at Unidade Local de Saúde de São José (ULS São José), Centro de Referência de Transplante Renal, Hospital Curry Cabral, Lisboa and included consecutive transplants performed, defined by the availability of both preservation fluid samples suitable for FTIR analysis and corresponding clinical outcome data. Sample processing and modeling were performed after outcome ascertainment to ensure that the study did not alter clinical management. Recipients subsequently provided written informed consent for research use of their clinical data and stored samples.

The primary endpoint was DGF, operationalized using the widely adopted dialysis-based definition (dialysis within the first 7 days post-transplant).

#### 2.1.2. Ethics Approval and Consent/Waiver

The study protocol was reviewed and approved by the ULS São José Ethics Committee (approval No. 1215/2022, 4 July 2022). Because this was a retrospective analysis of routinely collected clinical data and stored samples, and because all analyses were performed on de-identified data, written informed consent for the research use of clinical data and stored samples was obtained from recipients in accordance with institutional policy and applicable regulations. Any data extraction and handling followed institutional governance for patient confidentiality and research data protection.

#### 2.1.3. Eligibility Criteria (Inclusion/Exclusion) and Study Flow

**Figure 1:**
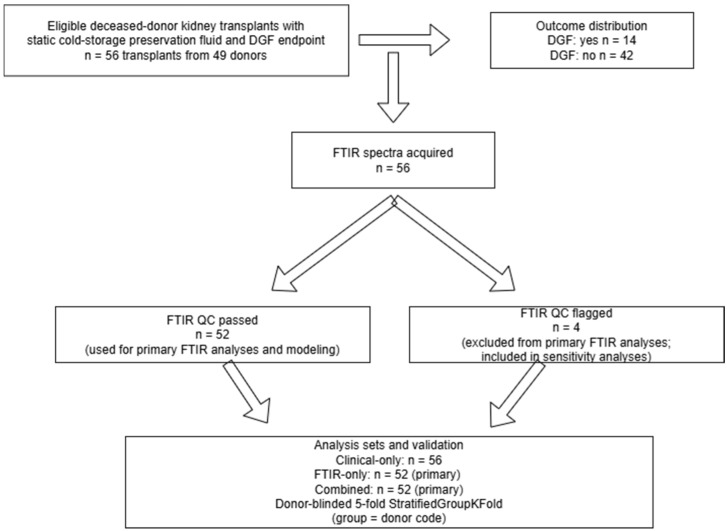
Cohort inclusion, FTIR quality control, and analysis sets.

Eligible cases were deceased donor kidney transplants for which (i) a preservation fluid sample was available for FTIR spectroscopic acquisition and (ii) DGF status (yes/no) could be ascertained from clinical records. Exclusion criteria for cohort inclusion were (i) missing primary endpoint information and (ii) insufficient sample volume or compromised sample integrity precluding FTIR acquisition. FTIR spectra failing predefined quality control criteria (e.g., major acquisition artifacts or outlier spectra that could not be corrected during preprocessing) were excluded from FTIR-containing analyses only; such cases remained eligible for the clinical-only analysis. Figure 1 summarizes the number of transplants included in the full clinical cohort, the QC-passed spectral dataset, and the corresponding analysis sets.

#### 2.1.4. Donor Pathways: DBD vs. DCD

Donor pathway was classified as donation after DBD or DCD donation according to the procurement documentation. DBD refers to organ donation after death determination by neurologic criteria (irreversible loss of brain function), whereas DCD refers to organ donation after death determination by circulatory/respiratory criteria (irreversible loss of circulatory function), typically following withdrawal of life-sustaining treatment in controlled DCD pathways. These pathways represent biologically distinct starting conditions for ischemia–reperfusion injury, with DCD kidneys commonly exposed to an additional period of warm ischemia before organ recovery [38,39].

In our controlled DCD pathway, the interval from circulatory arrest/death determination to initiation of organ recovery measures is protocol-limited to ≤15 min and includes a mandatory “no-touch” observation period of 10 min. In contrast, in DBD procurement, functional warm ischemia is minimized because organ cooling is initiated immediately after aortic cross-clamping and cold perfusion. Because the donor-level timestamps required to compute individual warm ischemia and cold ischemia times were not retrievable in this cohort, ischemia exposure was handled analytically via donor pathway (DBD vs. DCD) rather than as a continuous per-case variable.

#### 2.1.5. Preservation/Perfusion Solutions: Celsior^®^ vs. Custodiol^®^/HTK

Preservation solution was recorded as the solution used for organ flush and/or static cold storage and was categorized as Celsior^®^ or Custodiol^®^/HTK (histidine–tryptophan–ketoglutarate, HTK). Both solutions are established clinical preservation media but differ in composition (including buffering systems and electrolyte profiles), which can influence the biochemical “background” of preservation fluids and is therefore a relevant source of matrix variation for spectroscopy. Accordingly, solution type was treated as a structured variable and carried forward into stratified descriptions and robustness analyses to ensure that predictive performance was not driven by solution chemistry, rather than graft injury biology [29].

#### 2.1.6. Clinical Variables Collected (Donor/Recipient/Transplant)

Clinical variables were extracted from medical records and procurement documentation, focusing on information available before implantation to avoid post-outcome leakage. Donor variables included demographics (e.g., age and sex), donor pathway (DBD vs. DCD), terminal renal function markers when available (e.g., creatinine and related measures), and warm ischemia time (WIT) and CIT were not retrievable as individual-level variables in this cohort and therefore were not included among candidate predictors. Given the established influence of ischemia exposure on early graft dysfunction, donor pathway (DBD vs. DCD) and preservation solution type were retained as structured covariates and were used for stratified descriptions and robustness analyses. Recipient variables included demographics (e.g., age and sex), transplant history and baseline clinical descriptors available at the time of transplantation, and transplant-specific factors recorded in routine practice (e.g., immunologic matching summaries when available). These variables were used (i) for baseline cohort characterization and (ii) to build a prespecified clinical benchmark model against which FTIR-only and combined clinical plus FTIR models were compared. The purpose of this model hierarchy was to compare the predictive signal captured by routine clinical variables, FTIR spectra, and their combination under the same validation framework [1,2].

#### 2.1.7. Primary Endpoint Definition: Delayed Graft Function

DGF was the primary endpoint and was analyzed as a binary outcome (yes/no). DGF was defined as the requirement for at least one dialysis session within the first 7 days after transplantation, consistent with widely used definitions in the kidney transplant literature. DGF status was extracted from clinical records as a single indicator variable; information on dialysis indication, number of dialysis sessions, and DGF duration was not available in this dataset [1,15,40].

### 2.2. Preservation Fluid Sampling and Handling

#### 2.2.1. Sampling Timepoint and Workflow

Samples consisted of fluid obtained from the static cold storage solution at the time of organ preparation for transplant. For clarity, throughout this manuscript we refer to the sampled biofluid as static cold storage preservation fluid (not machine perfusion perfusate), reflecting that all grafts were stored in static cold conditions rather than on perfusion machines. Briefly, samples were collected at the time of transplantation upon graft arrival and prior to implantation, an aliquot of preservation fluid was obtained from the storage container/bag and transferred into pre-labeled tubes. The sampling workflow was standardized to minimize variability related to handling time, temperature, and potential contamination (e.g., avoiding visible blood clots or particulate material when possible). Donor pathway (DBD vs. DCD) and preservation solution type were recorded for each case, as these factors represent major sources of biological (injury phenotype) and analytical variability that could influence downstream spectral signatures and model behavior [26].

#### 2.2.2. Sample Processing, Aliquoting and Storage Conditions

Immediately after collection, samples were kept cold (on wet ice or at 4 °C) and processed as soon as feasible to reduce post-collection biochemical drift. Samples were gently mixed and, when visible debris was present, clarified by a brief centrifugation step (e.g., low-to-moderate g-force at 4 °C) to remove particulates that can introduce spectral artifacts and compromise reproducibility. The supernatant was then aliquoted into single-use cryovials and stored at −80 °C until FTIR analysis. Aliquoting was used to minimize repeated freeze–thaw cycles, which are widely recognized as a relevant pre-analytical variable capable of altering biomolecular and metabolite profiles. The number of freeze–thaw cycles was controlled by design (single thaw per aliquot whenever possible) [41,42,43].

For FTIR acquisition, aliquots were thawed on ice (or at 4 °C), gently mixed, and handled using a consistent preparation approach to reduce within-run variability. Pre-analytical discipline is particularly important for IR-based biofluid profiling, where the clinical value of the data is driven less by individual bands and more by reproducible sample preparation, quality control, and standardized preprocessing [23].

#### 2.2.3. Handling of Solution Matrix Effects (Pre-Analytical) (Celsior^®^ vs. HTK)

Because kidneys in this cohort were preserved in two clinically used solutions (Celsior^®^ and Custodiol^®^/HTK), solution type was treated as a structured source of matrix variation from the pre-analytical stage onward. Celsior^®^ and HTK differ substantially in composition (including buffering systems and osmotically active components), which can shift the baseline biochemical background of preservation effluent and, if ignored, can confound spectroscopic discrimination by allowing models to learn “solution identity” rather than graft injury biology.

Accordingly, pre-analytical handling was standardized across solutions (same storage temperature, aliquoting strategy, and thawing workflow), and analyses were designed to explicitly probe matrix effects: (i) solution type was retained as a metadata variable and used for stratified descriptions and robustness checks; (ii) when applicable, solution-specific blanks (unused Celsior^®^ and unused Custodiol^®^/HTK) were measured to characterize baseline spectral contributions from each matrix; and (iii) pooled quality control (QC) material was handled cautiously, avoiding cross-solution pooling when it could obscure matrix-specific behavior. While the comparative transplant literature suggests that clinical endpoints such as DGF may not differ markedly among commonly used static cold storage solutions in many settings, analytical equivalence cannot be assumed for spectroscopy, and explicit matrix control is therefore warranted. All QC metrics and exclusion flags were computed in Python as part of the reproducible analysis pipeline.

### 2.3. FTIR Spectroscopy Workflow

#### 2.3.1. FTIR Spectroscopy and Acquisition Settings

FTIR spectra were acquired following the same high-throughput transmission workflow previously established for kidney preservation fluids. Briefly, 25 μL aliquots were dispensed into a 96-well silicon microplate and dehydrated for approximately 3.5 h in a vacuum desiccator to reduce water-dominant interference and improve spectral reproducibility. Spectral data were collected using an FTIR spectrometer (Vertex 70, Bruker, Mannheim, Germany) equipped with a high-throughput screening accessory (HTS-XT, Bruker, Bruker, Mannheim, Germany) in transmission mode, spanning 400–4000 cm^−1^, with 32 co-added scans at 2 cm^−1^ resolution [22].

To minimize plate-related bias, sample placement was randomized and/or alternated across the plate to reduce spatial/edge effects, and sample identifiers were masked during preprocessing and downstream analyses. All spectra were acquired on the same instrument using identical parameters and environmental conditions whenever feasible.

Subsequent analyses were restricted to predefined spectral regions of interest: the fingerprint window (900–1800 cm^−1^) for primary modeling and the Amide I window (1600–1700 cm^−1^) for complementary protein-dominated signal evaluation.

#### 2.3.2. Spectral Preprocessing

Spectral preprocessing followed a fixed, standardized workflow consistent with our previously reported kidney preservation fluid FTIR pipeline. Atmospheric interference (H_2_O/CO_2_) was corrected in OPUS^®^ v6.5 (Bruker). Subsequent preprocessing was performed in Orange Data Mining (Bioinformatics Lab, University of Ljubljana, Ljubljana, Slovenia) using a locked sequence of operations, rubberband baseline correction, Savitzky–Golay first- and second-derivative transformations (third-order polynomial, 17-point window), and vector normalization. In parallel, equivalent steps and downstream data handling were reproduced in custom Python scripts (Python-based workflow) to enable full pipeline automation, leakage-controlled validation, and integration with the machine learning and clinical utility analyses.

#### 2.3.3. Quality Control (QC) Criteria and Outlier Handling

Spectral QC was conducted using a multi-metric framework adapted from our prior perfusate FTIR workflow, computed per sample spectrum (one spectrum per sample in this cohort). Four complementary QC metrics were used: (i) Amide I signal-to-noise ratio (SNR), defined as the maximum Amide I peak height (1600–1700 cm^−1^) after rubberband baseline correction divided by noise estimated in an off-band window (1800–1900 cm^−1^, with a predefined fallback window when required); (ii) spike artifact burden in the fingerprint region (900–1800 cm^−1^), detected via a first-difference approach and a median absolute deviation threshold (|z| > 6); (iii) cosine similarity between each vector-normalized fingerprint spectrum and the cohort median fingerprint spectrum as a measure of spectral-shape coherence; and (iv) baseline area fraction in the fingerprint region, defined as the area under the rubberband baseline divided by the raw spectral area as an index of baseline drift.

QC acceptability thresholds were reviewed a priori (plausible non-zero SNR, limited spike burden, high cosine similarity, and low baseline fraction). Spectra failing QC were flagged for review and excluded from downstream modeling when criteria indicated substantial acquisition/preparation artifacts. Because two preservation solutions (Celsior^®^ and Custodiol^®^/HTK) were present, QC distributions were also inspected stratified by solution to ensure that exclusion decisions reflected true spectral quality issues rather than solution-specific matrix background. Accordingly, FTIR-only and combined primary analyses were performed on the QC-passed spectral dataset, whereas the clinical-only model used the full cohort with available clinical outcome data.

#### 2.3.4. Exploratory Unsupervised Analysis

The unsupervised analyses were performed based on cosine distance multidimensional scaling (MDS) and Euclidean distance; Ward linkage hierarchical clustering (HCA) was performed to examine the cohort structure and screen outliers [44].

The t-distributed stochastic neighbor embedding (t-SNE) was applied to explore local neighborhood structure in the high-dimensional spectral space. Because t-SNE embeddings can be sensitive to hyperparameters and random initialization, the results were interpreted cautiously and used only to support qualitative visualization rather than infer separation strength [45].

#### 2.3.5. Supervised Analysis Overview

Supervised classification was conducted to predict the clinical endpoint using a pre-specified pipeline consistent with our earlier perfusate FTIR study; in particular, preprocessing and region windowing were followed by feature selection using the fast correlation-based filter (FCBF) and classification using simple, interpretable learners (e.g., Naïve Bayes).

Feature selection and model training were implemented under leakage-controlled validation (described in detail in the machine learning section), and model outputs were evaluated using discrimination and calibration metrics; clinical utility was assessed using decision-analytic methods in the validation framework.

### 2.4. Prediction Modeling Strategy

To compare the predictive information captured by FTIR spectra, conventional pre-implant clinical variables, and their combination, we prespecified a three-model strategy: (i) a clinical baseline model (Model 1), (ii) a spectroscopy-only model (Model 2), and (iii) an integrated clinical plus FTIR model (Model 3) [46]. All predictors were restricted to information available before implantation to avoid post-outcome leakage. Because the dataset includes paired kidneys from the same donor, all primary model development and internal validation were performed using donor-grouped cross-validation to avoid information leakage. Analyses performed without donor grouping are presented only as exploratory “optimistic” estimates for transparency.

#### 2.4.1. Model 1: Clinical Baseline Model

Model 1 served as the prespecified benchmark and included only clinical variables available at or before implantation. Candidate predictors were selected a priori based on established DGF risk modeling and routine availability in transplant workflows, including donor age, donor pathway (DBD vs. DCD), terminal donor renal function markers (e.g., donor creatinine, when available), and other routinely available pre-implant descriptors. Individual cold and warm ischemia times were not available in this cohort and were therefore not included, and basic donor/recipient descriptors (e.g., sex and recipient age, when available). Where a composite donor risk score (e.g., KDPI (Kidney Donor Profile Index) and KDRI (Kidney Donor Risk Index)) was available, it was considered as an alternative representation of donor risk to avoid duplicating correlated inputs; the final clinical feature set was prespecified before model training and applied consistently in validation.

#### 2.4.2. Model 2: FTIR-Only Model

Model 2 used FTIR spectra exclusively. Spectral predictors were derived from the predefined fingerprint region (900–1800 cm^−1^) after spectra preprocessing (baseline correction, Savitzky–Golay derivatives, and vector normalization), following the core workflow established in our prior kidney perfusion fluid FTIR study. To control dimensionality and reduce redundancy in the high-dimensional spectral feature space, feature selection was performed using the FCBF, an information-theoretic filter that retains relevant features while removing redundant ones.

Given the modest sample size and the aim of interpretability and robustness, we prioritized simple classifiers with probabilistic outputs, with Naïve Bayes as the primary learner. This model choice was intended to reduce overfitting risk while still capturing the dominant discriminatory structure in the spectral data.

#### 2.4.3. Model 3: Combined Clinical Plus FTIR Model

Model 3 represented the final integrated predictor and combined pre-implant clinical predictors (Model 1 feature set) with the FTIR-derived information from Model 2. Integration was implemented using an additive feature strategy in which selected FTIR features (after FCBF) were concatenated with the clinical variables and fitted within the same supervised framework used for the clinical model. In addition, as a stability-focused sensitivity approach, we considered an “FTIR score” representation (the cross-validated predicted probability from Model 2) as a single spectral summary feature to be combined with the clinical predictors, reducing the degrees of freedom while preserving the spectral signal.

Across all models, primary internal validation was performed using donor-blinded 5-fold StratifiedGroupKFold cross-validation, with Donor Code used as the grouping variable. Within each training fold, preprocessing, encoding, imputation, and FTIR feature selection were fitted using the training data only and then applied to the corresponding held-out fold. Pooled out-of-fold predictions were then used to assess discrimination, calibration, and clinical utility, as described below.

### 2.5. Feature Selection and Leakage Control

#### 2.5.1. Feature Selection

To control dimensionality and redundancy in the high-dimensional FTIR feature space, we used the FCBF, an information-theoretic filter that identifies relevant predictors while removing redundant features. Crucially, feature selection was performed only on the training data within each resampling iteration, ensuring that the validation folds were never used to select or rank spectral variables and preventing optimistic bias from information leakage [47,48].

#### 2.5.2. Encoding/Scaling/Imputation Strategy (Performed Within Folds)

All data transformations were implemented under strict leakage control. Categorical clinical variables were encoded within the training folds, and continuous predictors were standardized when required by the learning algorithm. Missing predictor values, when present, were imputed using parameters estimated from the training data only and then applied to the corresponding held-out fold. This fold-wise preprocessing strategy was applied consistently across Model 1 (clinical), Model 2 (FTIR-only), and Model 3 (combined), so that performance estimates reflect true out-of-sample behavior rather than preprocessing artifacts.

Analyses were carried out using Orange Data Mining and reproducible Python scripts (for automated, fold-aware pipelines and downstream validation/utility calculations).

#### 2.5.3. Class Imbalance Handling (Class Weights/Resampling; PR-AUC Rationale)

Given the expected imbalance in DGF prevalence, we complemented ROC-based discrimination with precision–recall analysis (area under the precision–recall curve (PR-AUC)), which is often more informative when the positive class is less frequent [49].

During model training, class imbalance was addressed using class-weighted learning (and, where applicable, sensitivity analyses using resampling strategies) applied within the training folds only.

#### 2.5.4. Donor-Blinded Analysis Workflow

To make the leakage control structure explicit, the donor-blinded primary analysis proceeded as follows: (1) the dataset was split using 5-fold StratifiedGroupKFold, with Donor Code used as the grouping variable so that paired kidneys from the same donor were always assigned to the same fold; (2) within each training fold, categorical encoding, imputation, and scaling were fitted using the training data only; (3) for FTIR-containing models, FTIR feature selection was also performed using the training data only; (4) the fitted fold-specific transformations were then applied to the corresponding held-out fold; (5) the model was trained on the transformed training data and used to generate predictions for the held-out fold; and (6) out-of-fold predictions from all folds were pooled to estimate ROC-AUC, PR-AUC, Brier score, calibration, and decision curve analysis. No data-driven preprocessing or feature-selection step was fitted on the full dataset before donor-blinded validation.

### 2.6. Validation Design and Performance Assessment

#### 2.6.1. Internal Validation and Resampling (Donor-Blinded)

Internal validation was implemented in Python using StratifiedGroupKFold (5-fold), with Donor Code as the grouping variable to prevent donor-level leakage while preserving the DGF class distribution across folds. All preprocessing and feature selection steps (including FCBF) were fitted only on the training split of each fold and then applied to the corresponding held-out fold. Out-of-fold predictions from all folds were pooled for discrimination, calibration, and decision-analytic evaluation. This donor-grouped 5-fold scheme was the prespecified primary internal validation procedure for the three main models.

#### 2.6.2. Discrimination Metrics

Model discrimination was assessed using the area under the receiver operating characteristic curve (ROC-AUC) and, given imbalance considerations, the PR-AUC [50].

Threshold-based metrics (sensitivity, specificity, and balanced accuracy) were computed using decision thresholds determined within training folds only (e.g., Youden-based or clinically prespecified) and then applied to corresponding held-out predictions to avoid optimistic thresholding. Model discrimination was assessed using ROC-AUC and, given class imbalance, PR-AUC, computed as average precision from out-of-fold prediction scores.

#### 2.6.3. Calibration Assessment (Brier Score, Calibration Curve; Slope/Intercept if Desired)

Because clinical decision support depends on well-calibrated probabilities, we evaluated calibration using calibration curves and the Brier score as a measure of overall probabilistic accuracy [51].

Calibration assessment and Brier scores were computed from out-of-fold predicted data (outer-loop predictions) to reflect validated probability performance [48].

#### 2.6.4. Robustness Analysis

Pre-specified robustness analyses examined whether model performance was consistent across key sources of heterogeneity: (i) Donor pathway: DBD vs. DCD (subgroup performance and descriptive calibration); (ii) Preservation solution: Celsior^®^ vs. Custodiol^®^/HTK (subgroup performance and QC distribution checks).

### 2.7. Clinical Utility

#### Decision Curve Analysis (DCA)

Clinical utility was evaluated using DCA, which estimates net benefit across threshold probabilities and compares model-guided strategies with default approaches (“treat all” and “treat none”). DCA was performed using validated, out-of-fold predicted probabilities from the outer loop to avoid optimistic bias, and interpretation/reporting followed established guidance [35,37]. DCA was implemented in Python using out-of-fold predicted probabilities from StratifiedGroupKFold.

### 2.8. Reporting, Risk of Bias, and Reproducibility

#### 2.8.1. Reporting Guideline: TRIPOD+AI

Model development, validation, and reporting were structured according to the TRIPOD+AI recommendations for prediction model studies using machine learning methods. In this study, we report cohort characteristics and outcome prevalence, candidate predictors and their availability at the time of prediction, missing data handling, all preprocessing and feature selection steps, the donor-grouped 5-fold internal validation strategy, and model performance in terms of discrimination, calibration, and clinical utility [32]. A completed TRIPOD+AI checklist is provided as Supplementary File S1.

#### 2.8.2. Risk of Bias/Applicability: PROBAST+AI

Potential risks of bias and applicability concerns were considered using the PROBAST+AI framework, with attention to participant selection, outcome definition, predictor handling, and analysis choices that can inflate apparent performance [34]. In this study, the main anticipated risks of bias were the small sample size relative to predictor dimensionality, the absence of external validation, the inability to include case-level CIT and WIT, and the potential for preservation solution confounding in FTIR-based models. Applicability is also limited by the single-center design and the use of a pragmatic DGF endpoint that may vary across centers. These considerations informed our decision to frame the study as proof-of-concept, rather than as a clinically deployable model.

#### 2.8.3. Software, Versions, and Code Availability

Spectral preprocessing and exploratory analyses were performed using Orange Data Mining v3.39 (visual workflow) alongside a reproducible Python v3.14.0 implementation of the same pipeline to enable fold-aware preprocessing, donor-grouped internal validation, and downstream evaluation. Acquisition-related preprocessing steps (e.g., atmospheric correction) were performed in OPUS^®^ (Bruker). The computational pipeline was executed with fixed random seeds where applicable (e.g., for t-SNE visualizations). In the primary analysis, all data-driven steps, including FTIR feature selection, encoding, standardization, and imputation, were performed within each training fold of donor-grouped 5-fold StratifiedGroupKFold cross-validation to prevent information leakage. Software versions and key package dependencies were recorded as part of the reproducible analysis environment and will be provided with the released analytical code.

Because FTIR feature selection was performed within training folds and the present study was limited to internal validation, we do not claim a single locked final deployable model in this manuscript. Instead, we report the prespecified model classes, predictor domains, preprocessing steps, feature selection procedure, and donor-blinded validation framework in sufficient detail to support reproducibility of the analytical strategy. A fixed final model specification intended for clinical deployment will require retraining under a locked pipeline in a larger dataset followed by external validation and recalibration.

The analytical Python code used for donor-blinded preprocessing, feature selection, model training, and validation is not publicly posted at this stage but will be made available upon acceptance, with public release through a repository or, where required, through academic access arrangements subject to institutional approval.

## 3. Results

### 3.1. Study Population, Sample Flow, and Endpoint Distribution

A total of 56 kidney transplants were included. These transplants originated from 49 deceased donors, because 7 donors contributed both kidneys to two different recipients. DGF occurred in 14/56 (25.0%) transplants, while 42/56 (75.0%) had no DGF (Table 1). This structure implies that some donor-level characteristics are duplicated across paired kidneys from the same donor, and this donor pairing was considered when interpreting unadjusted comparisons [31,32].

In unadjusted baseline comparisons (Table 1), DGF was strongly associated with donation after circulatory death (DCD): 10/14 (71.4%) in the DGF group versus 3/42 (7.1%) in the no-DGF group (*p* < 0.001, Fisher’s exact test). Donor renal function indicators were less favorable among grafts with DGF, including higher donor serum creatinine (median 1.09 [IQR 0.97–1.47] vs. 0.80 [0.60–1.05] mg/dL; *p* = 0.008, Mann–Whitney U test) and lower donor eGFR (69.79 ± 24.32 vs. 90.86 ± 29.73 mL/min/1.73 m^2^; *p* = 0.013, Welch’s *t*-test). Donor risk indices were also higher in the DGF group: KDPI 64.54 ± 25.23 vs. 46.93 ± 25.81 (*p* = 0.041) and KDRI 1.20 ± 0.31 vs. 0.99 ± 0.26 (*p* = 0.044) (both by Welch’s *t*-test; KDPI/KDRI available for 54/56 transplants). Recipient age was modestly higher in the DGF group (*p* = 0.046), whereas other recipient baseline characteristics were broadly comparable between groups (Table 1).

### 3.2. FTIR Spectral Data Quality Control and Retained Spectral Dataset

All 56 atmospheric-compensated raw FTIR spectra were subjected to an a priori QC workflow focused on the spectral windows that concentrate the most relevant biochemical information in biofluids; namely, the fingerprint region (900–1800 cm^−1^) and the protein-dominated Amide I band (1600–1700 cm^−1^) [22,52].

Four complementary QC metrics were computed per spectrum, consistent with established biofluid FTIR pipelines: (i) Amide I SNR, calculated as the maximum Amide I peak height after rubberband baseline correction divided by the standard deviation in an off-band noise window (1800–1900 cm^−1^; 2000–2200 cm^−1^ as a predefined fallback); (ii) spike artifact burden in the fingerprint region detected on the first-difference derivative using a robust MAD-based threshold; (iii) cosine similarity between each vector-normalized fingerprint spectrum and the cohort median fingerprint spectrum as a spectral shape coherence index; and (iv) baseline area fraction in the fingerprint window (rubberband baseline area divided by raw spectral area) as a drift index [22,53].

**Figure 2:**
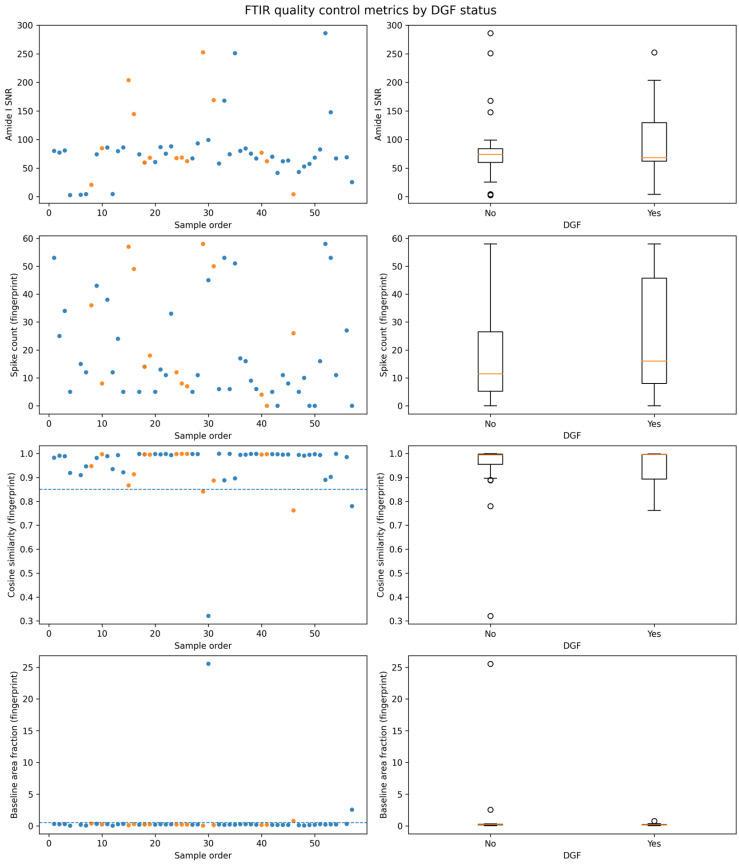
FTIR spectral quality control metrics by delayed graft function status. (**Left**) panels: per-spectrum QC metrics plotted by acquisition/sample order, with points colored by DGF status (Orange DGF and blue non-DGF). (**Right**) panels: distribution of the same metrics by DGF group (boxplots show median and interquartile range; yellow line indicate 1.5 × IQR; circle denote outliers). Metrics include Amide I signal-to-noise ratio (1600–1700 cm^−1^), spike count in the fingerprint region, cosine similarity to the cohort median fingerprint spectrum (shape coherence index), and baseline area fraction in the fingerprint region. Dashed horizontal lines indicate conservative outlier flag thresholds (cosine similarity < 0.85; baseline fraction > 0.50), used for QC flagging prior to downstream analyses.

Overall QC behavior indicated analytically usable spectra across the cohort (Supplementary Table S1; Figure 2). Median [IQR] values were 71.92 [60.26–84.97] for Amide I SNR, 12.0 [6.0–33.25] for spike count, 0.994 [0.931–0.997] for cosine similarity, and 0.205 [0.152–0.250] for baseline fraction. Importantly, none of the QC metrics showed evidence of systematic differences by clinical endpoint: two-sided Mann–Whitney U tests comparing DGF (yes, *n* = 14) versus no DGF (*n* = 42) were non-significant for SNR (*p* = 0.762), spike count (*p* = 0.329), cosine similarity (*p* = 0.791), and baseline fraction (*p* = 0.623) (Table 2), arguing against a technical–quality imbalance confounding downstream comparisons.

**Figure 3:**
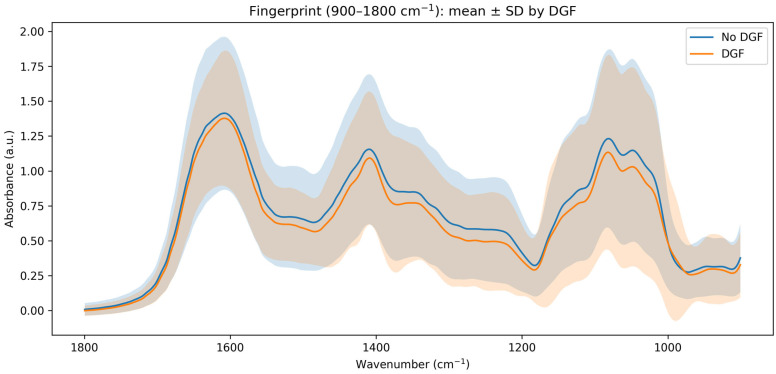
Fingerprint region mean spectrum (±SD) by DGF status. Mean atmospheric-compensated raw absorbance spectra in the fingerprint region (900–1800 cm^−1^) for DGF No and DGF Yes groups. Shaded bands indicate ±1 standard deviation (SD) across spectra within each group.

Using conservative, pre-specified outlier flags (cosine similarity <0.85 and/or baseline fraction >0.50), 4/56 spectra (7.1%) were flagged for review (samples 29, 30, 46, and 57). The flags reflected either markedly reduced spectral shape coherence (low cosine similarity) or pronounced baseline dominance in the fingerprint region (high baseline fraction), consistent with acquisition-related artifacts rather than endpoint-related effects (Figure 3). The primary FTIR-only and combined analyses therefore used the QC-passed spectral dataset (*n* = 52), whereas the clinical-only model remained based on all 56 transplants. All key FTIR-containing results were additionally repeated in a sensitivity analysis including the flagged spectra (*n* = 56) to assess robustness.

### 3.3. Global Spectral Structure and Potential Confounding

To explore the global structure of the dataset and identify potential technical drivers of variance before supervised modeling, we performed an unsupervised principal component analysis (PCA) focused on the biofingerprint region (900–1800 cm^−1^), which concentrates much of the diagnostically relevant biochemical information in biofluids. Spectra passing QC were clipped to this region and preprocessed using a Savitzky–Golay second derivative (17-point window, third-order polynomial) followed by vector normalization to emphasize band shape differences while reducing baseline/scatter effects.

In the QC-passed dataset (*n* = 52 spectra), the first two principal components explained 38.1% (PC1) and 27.1% (PC2) of the total variance (cumulative 65.2%). The PCA score space did not show a distinct separation between DGF (yes) and no DGF spectra, which largely overlapped across PCs, consistent with the absence of endpoint-related technical bias observed in the QC metrics (Table 2). By contrast, the preservation solution (Celsior^®^ vs. Custodiol^®^/HTK) emerged as a major driver of spectral variance, primarily along PC1 (solution-associated differences in PC1 scores; Mann–Whitney U *p* = 0.00045). These findings indicate that broad spectral variability is dominated by non-endpoint technical/processing factors rather than by DGF status, and they support explicit accounting for preservation solutions in subsequent supervised models. They also reinforce that solution-related variance is a genuine source of confounding in this dataset and should be treated as a central design consideration in future validation studies.

**Figure 4:**
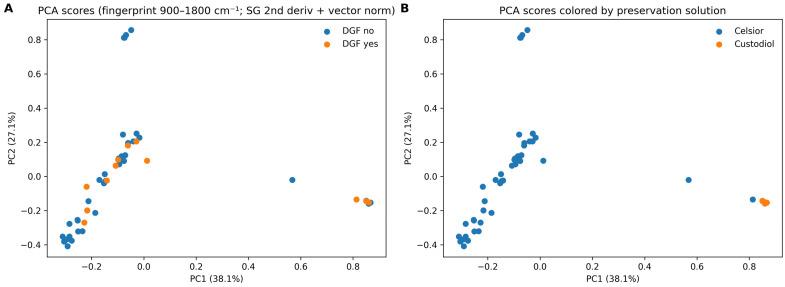
Principal component analysis (PCA) of QC-passed fingerprint spectra (900–1800 cm^−1^). (**A**) Scores colored by delayed graft function (DGF) status. (**B**) The same scores colored by preservation solution (Celsior^®^ vs. Custodiol^®^/HTK).

Consistent with the PCA results, group mean spectra in the fingerprint ROI were broadly similar between DGF groups (Figure 4), with only subtle, localized differences within the carbohydrate/phosphate subregion (~1050 cm^−1^) and within protein-associated bands in the higher fingerprint range. These differences were small relative to within-group variability (standard deviation envelopes), reinforcing that any DGF-associated signal, if present, is likely modest and must be evaluated using appropriately regularized supervised models and sensitivity analyses rather than inferred from unsupervised clustering alone.

**Figure 5:**
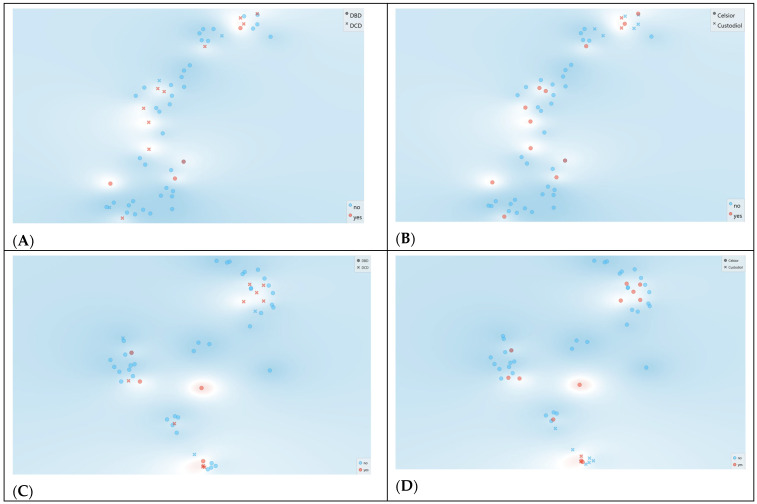
t-SNE embeddings of FTIR fingerprint spectra under alternative feature representations. (**A**) Full fingerprint feature set, points colored by DGF status and shaped by donor type (DBD vs. DCD). (**B**) Full fingerprint feature set, points colored by DGF status and shaped by preservation solution (Celsior^®^ vs. Custodiol^®^/HTK). (**C**) Reduced spectral representation using a ranked subset of wavenumber features, colored by DGF status and shaped by donor type. (**D**) Reduced spectral representation, colored by DGF status and shaped by preservation solution. Each point represents one preservation fluid sample. t-SNE is shown as an exploratory visualization of local neighborhood structure and does not imply global class separability.

Exploratory visualization of FTIR spectral data using t-distributed stochastic neighbor embedding (t-SNE) did not reveal distinct clusters separating DGF vs. non-DGF samples, although local regions of increased DGF density were observed (Figure 5). Similar projections colored by donor type (DBD vs. DCD) and by preservation solution (Celsior^®^ vs. Custodiol^®^/HTK) suggested that donor and solution effects contribute to spectral variance, but no simple two-dimensional embedding was sufficient to clearly segregate outcome groups.

**Figure 6:**
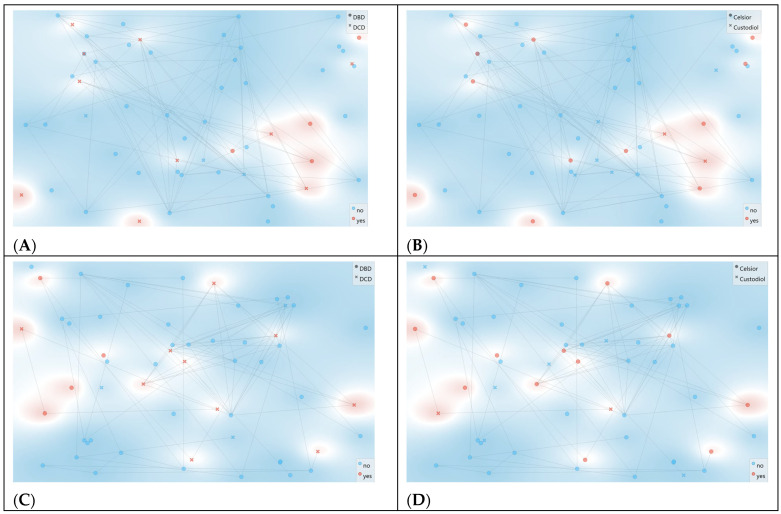
MDS projections of FTIR fingerprint spectra (full vs. ranked feature sets). Two-dimensional metric MDS projections computed from pairwise spectral distances after the same preprocessing applied to the FTIR fingerprint data. Points are colored by delayed graft function (DGF; blue = no, red = yes). (**A**) Full FTIR fingerprint feature set; point shape indicates donor type (DBD vs. DCD). (**B**) Full FTIR fingerprint feature set; point shape indicates preservation solution (Celsior^®^ vs. Custodiol^®^/HTK). (**C**) Reduced (“ranked”) FTIR feature set; point shape indicates donor type (DBD vs. DCD). (**D**) Reduced (“ranked”) FTIR feature set; point shape indicates preservation solution (Celsior^®^ vs. Custodiol^®^/HTK).

MDS projections further confirmed the absence of sharply separated clusters by outcome or by preservation fluid (Figure 6), consistent with a high-dimensional signal structure rather than a few dominant axes of separation.

**Figure 7:**
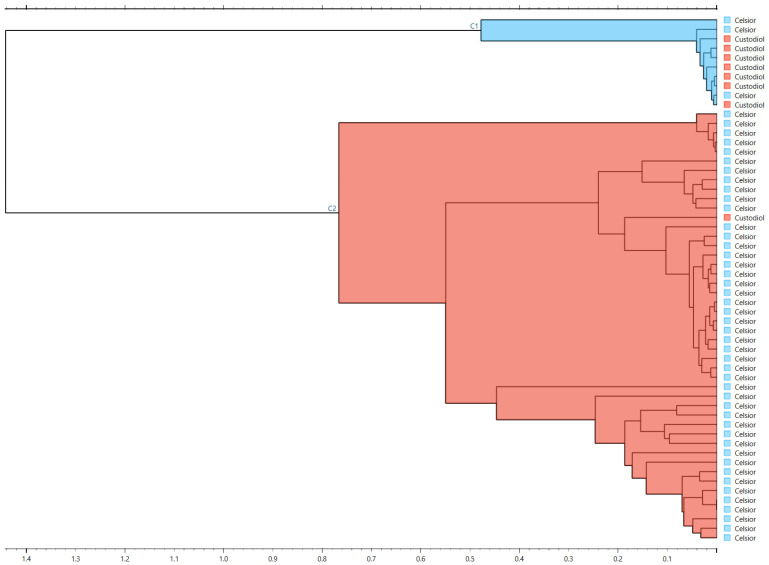
Hierarchical clustering of FTIR fingerprint spectra reveals a dominant preservation solution partition. Dendrogram obtained using correlation-based distances (1—Pearson correlation) and average linkage on normalized fingerprint region spectra. Leaf labels indicate preservation solution (Celsior^®^ vs. Custodiol^®^/HTK).

Hierarchical clustering of spectral distances (Euclidean distance; Ward linkage) revealed broad grouping primarily influenced by preservation fluid type rather than DGF status, with one dominant cluster comprising predominantly Celsior^®^ samples and a separate cluster enriched for Custodiol^®^/HTK (Figure 7). These exploratory analyses support the notion that the DGF signal in FTIR profiles is subtle and embedded in multivariate structure, motivating the predictive modeling approach presented below.

Given the strong solution-associated structure observed in unsupervised analyses, subsequent predictive modeling explicitly evaluated performance under donor-grouped validation to mitigate donor-level and matrix-related confounding.

### 3.4. Prediction Models and Discrimination Performance

To compare the predictive signal captured by routine clinical variables, FTIR spectra, and their combination, we evaluated three model families: (i) clinical-only models, (ii) FTIR-only models, and (iii) combined clinical–FTIR models. We report two complementary analyses. First, we summarize exploratory benchmark analyses obtained under standard stratified cross-validation; these analyses treat each transplant as an independent observation and are presented for descriptive comparison only. Second, because some donors contributed two kidneys, the prespecified primary internal validation uses donor-grouped cross-validation (grouped by Donor Code) to avoid optimistic estimates due to shared donor-level characteristics. Unless stated otherwise, the main interpretation is based on the donor-grouped analysis. Because of the modest sample size and sparse events in several strata, subgroup benchmark analyses were retained only for descriptive transparency and are reported in the Supplementary Materials. These subgroup estimates were highly unstable and should not be interpreted as primary evidence of model performance. The primary interpretation of model performance is based on the donor-grouped full-cohort analysis reported in Section 3.5.

#### 3.4.1. Clinical-Variable Benchmark Models

Clinical-only benchmark models for delayed graft function (DGF; yes/no) using routinely available donor- and recipient-level variables were implemented. Two complementary learners were evaluated: a classification tree and Naïve Bayes. Model performance metrics are summarized in Supplementary Table S2, as described in Materials and Methods.

To reduce redundancy among clinical predictors, we applied FCBF and compared models trained on all variables versus an FCBF-selected subset; the complete ranked list and selected predictors are provided in Supplementary Table S3.

In addition, given the well-known clinical heterogeneity between DBD and DCD grafts, we report results not only for the full cohort but also stratified by donor type (DCD-only and DBD-only) to explicitly quantify performance heterogeneity across these clinically distinct settings (Supplementary Table S2).

##### Full Cohort (DBD + DCD)

Across the full cohort, applying FCBF feature selection generally improved the clinically relevant sensitivity–specificity balance compared with using all variables. In particular, the FCBF-selected Naïve Bayes benchmark achieved high overall accuracy (CA 0.875) with good discrimination (AUC 0.863 when DGF = yes was set as target class) and a favorable trade-off between sensitivity for DGF (recall 0.714) and specificity (0.929) (Supplementary Table S2). The FCBF-selected tree showed similar accuracy (CA 0.857) but lower sensitivity (recall 0.571), despite very high specificity (0.952), consistent with a more conservative classifier that prioritizes avoiding false positives at the expense of missing some DGF cases (Supplementary Table S2). These results support using the FCBF-selected clinical benchmark as the primary clinical reference for subsequent comparisons against FTIR-only and combined models.

##### Donor Type (DCD-Only and DBD-Only)

Within donor type strata, performance estimates became substantially more variable because of the small sample sizes and sparse outcome counts; these analyses are therefore informative mainly as illustrations of instability rather than as robust subgroup performance estimates. In the DCD-only subset (*n* = 14), class-specific AUC values were reported as NA under 5-fold cross-validation in some runs (Supplementary Table S2), reflecting folds where one of the outcome classes was absent, which makes metrics such as AUC undefined.

For interpretability, we report DCD-only class-specific results under a lower-fold stratified setting for the FCBF-selected models, yielding defined AUCs and more stable class-specific behavior (Supplementary Table S2). In this small subset, the FCBF-selected Naïve Bayes model favored sensitivity (recall 1.000 for DGF = yes) with moderate specificity (0.600), whereas the corresponding tree model showed lower sensitivity (Supplementary Table S2). These DCD-only estimates should be interpreted cautiously and primarily as an exploration of subgroup behavior rather than as definitive performance claims.

In the DBD-only subset (*n* = 42), the impact of feature selection was again evident. Models trained on all clinical variables showed unstable class-specific behavior, consistent with class imbalance in this stratum. In contrast, FCBF-selected Naïve Bayes achieved the strongest discrimination (AUC 0.910 for DGF = yes) with moderate accuracy (CA 0.721) and a balanced sensitivity–specificity profile (recall 0.750; specificity 0.718) (Supplementary Table S2). Overall, these findings indicate that (i) feature selection improves robustness in this cohort and (ii) clinical model performance is not uniform across donor type strata, reinforcing the need to evaluate FTIR-only and combined models under the same stratified lens.

#### 3.4.2. FTIR-Only Benchmark Models

To assess whether the FTIR spectral fingerprint alone contains predictive information for DGF, we trained benchmark classifiers using only the FTIR-derived features (preprocessing fixed a priori; Savitzky–Golay window length 17). Model performance was estimated using stratified 2-fold cross-validation, selected to preserve class representation in the test folds given the limited cohort size. These exploratory benchmark FTIR-only analyses were run on the full available benchmark dataset, whereas the prespecified primary FTIR-containing analyses reported in Section 3.5 used the QC-passed spectral dataset.

Because donor pathway and preservation/perfusion solution may influence the biochemical composition of perfusate and therefore the infrared signature, we evaluated FTIR-only models not only in the full cohort (All donors, *n* = 56) but also in donor type × perfusion solution strata: DBD + Celsior^®^ (*n* = 36), DBD + Custodiol^®^/HTK (*n* = 6), DCD + Celsior^®^ (*n* = 12), and DCD + Custodiol^®^/HTK (*n* = 2). These subgroup analyses were intended as sensitivity analyses to probe heterogeneity and potential confounding by solution. Given the very small stratum sizes in some donor type × solution subsets, these analyses were retained only to illustrate heterogeneity and instability and should not be interpreted as standalone evidence of subgroup performance.

In the full cohort, FTIR-only modeling using all FTIR features yielded poor discrimination (AUC 0.443; CA 0.304), with a strong imbalance between sensitivity and specificity depending on the positive class definition. To reduce redundancy among spectral predictors, we applied FCBF and re-trained the same benchmark models on the reduced FTIR feature set. With FCBF-selected FTIR predictors, discrimination improved substantially (average AUC 0.849; CA 0.804), with sensitivity 0.857 and specificity 0.786 when “DGF = yes” was treated as the positive class.

Across donor type × solution strata, performance estimates varied markedly (Supplementary Table S4). In the DBD + Celsior^®^ subset, FCBF produced high AUC values (average AUC 0.946; AUC 0.978 for DGF = yes), but classification accuracy remained low (CA 0.216) with extreme class-wise behavior (sensitivity 1.000 and specificity 0.147 for DGF = yes), consistent with unstable decision thresholds in an imbalanced setting. In the DCD + Celsior^®^ subset, FCBF also yielded high AUC (average AUC 0.861; AUC 0.950 for DGF = yes), but accuracy was moderate (CA 0.545) and sensitivity/specificity traded off sharply (sensitivity 0.444; specificity 1.000 for DGF = yes).

Two strata were too small for stable inference. In DBD + Custodiol^®^/HTK, reported AUC as undefined (“NA”) for class-specific settings because some test folds contained only one outcome class, a known limitation of AUC estimation under very small, stratified splits; accordingly, sensitivity/specificity became degenerate (0.000 or 1.000). In DCD + Custodiol^®^/HTK, cross-validated estimates are not meaningful (AUC/CA near 0.0), and this subset should be considered non-evaluable for model performance claims.

Overall, FTIR-only models showed limited predictive value without feature reduction, while FCBF-based spectral compression increased apparent discrimination in the overall cohort. Subgroup analyses suggested strong instability and non-generalizability of thresholds in small strata, reinforcing that solution- and donor-related heterogeneity must be accounted for and that subgroup results should be interpreted as exploratory.

Having established separate performance profiles for clinical-only and FTIR-only models, we next evaluated integrated models that jointly leverage clinical predictors and spectral information to assess whether FTIR contributes incremental predictive value for delayed graft function.

#### 3.4.3. Combined Clinical and FTIR Model Performance

To assess whether FTIR spectral data adds predictive information to routinely available clinical variables, we trained combined (“mixed”) models in which clinical predictors and FTIR spectral features were jointly used to predict DGF. Because the combined feature space is high-dimensional and contains correlated predictors, we also evaluated a filter-based feature reduction using the FCBF. These exploratory combined model benchmark analyses were run on the full available benchmark dataset, whereas the prespecified primary FTIR-containing analyses reported in Section 3.5 used the QC-passed spectral dataset.

Performance was assessed using stratified cross-validation in Orange (Test and Score). We used 5-fold cross-validation when feasible and reduced to 3-fold in analyses that produced very small strata (e.g., donor type subsets) to preserve evaluable folds, reporting weighted average metrics over classes (Target class = “(None)”) and class-specific metrics when selecting “DGF yes” or “DGF no”.

In the overall cohort (merged dataset *n* = 56), the combined Naïve Bayes model achieved an AUC of 0.782, with accuracy 0.873, recall 0.873, and specificity 0.768 (Supplementary Table S5). Applying FCBF feature selection improved discrimination and overall performance (AUC 0.887, accuracy 0.891, recall 0.891, specificity 0.822), supporting the idea that a reduced mixed feature subset (clinical + selected spectral variables) may better capture clinically relevant signal while mitigating noise and redundancy.

When models were evaluated by donor type, performance differed substantially. In DCD-only cases (*n* = 14), the combined model reached AUC 0.733 with accuracy/recall 0.692 and specificity 0.908 (Supplementary Table S5), but this estimate should be interpreted cautiously given the small sample size. In DBD-only cases (*n* = 42), the combined model without feature reduction showed poor average performance (AUC 0.500; accuracy/recall 0.095), whereas the FCBF-reduced combined model showed very high apparent performance (AUC 0.980; accuracy/recall 0.952; specificity 0.995).

This strong sensitivity to feature reduction and stratification is consistent with well-known small-sample/high-dimensional behavior, where cross-validation estimates can become unstable when folds are not fully representative (including the risk of undefined or inflated metrics in extreme imbalance scenarios). Accordingly, donor type–stratified combined model results are best viewed as exploratory and should be confirmed using stricter validation strategies (e.g., donor-grouped splits) and larger external cohorts. The donor type–stratified combined model benchmark estimates were highly unstable and are presented for exploratory transparency only; the primary interpretation of combined model performance is based on the donor-blinded grouped analysis in Section 3.5.

### 3.5. Calibration and Clinical Usefulness

Calibration and potential clinical usefulness were evaluated using out-of-fold predicted probabilities for the clinical-only, FTIR-only, and combined models. Because two kidneys from the same donor may contribute to the dataset, we prioritized a donor-blind internal validation in which all transplants sharing the same Donor Code were kept within the same fold (thus preventing leakage between training and testing). This approach reduces optimistic performance estimates driven by within-donor correlation.

#### 3.5.1. Calibration (Donor-Blinded; Grouped CV by Donor Code)

**Figure 8:**
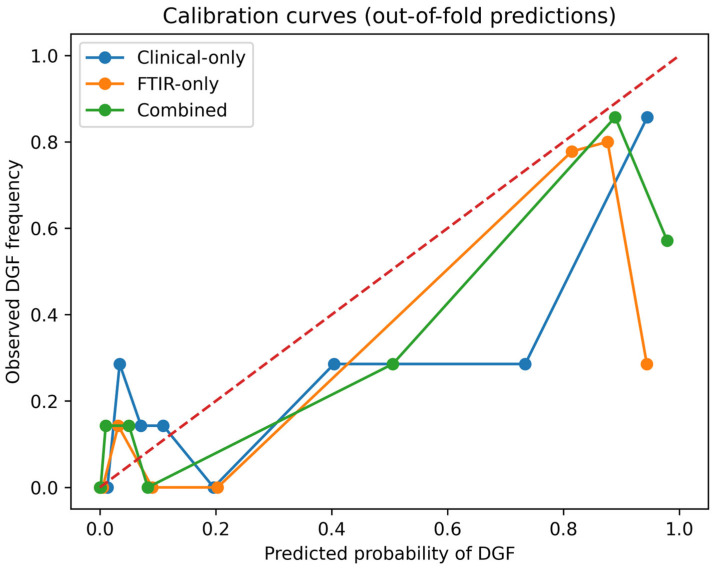
Calibration curves for DGF prediction models using donor-grouped out-of-fold probabilities. Calibration curves for clinical-only, FTIR-only, and combined models based on out-of-fold predicted probabilities from StratifiedGroupKFold (5-fold) using Donor Code as the donor grouping variable.

Using StratifiedGroupKFold (5-fold) with Donor Code as the grouping variable, donor-blinded discrimination (out-of-fold ROC-AUC) was 0.775 for the clinical-only model, 0.814 for the FTIR-only model, and 0.796 for the combined model. Probabilistic accuracy, assessed with the Brier score (lower values indicate better overall probability accuracy), was 0.162 for the clinical-only model, 0.194 for the FTIR-only model, and 0.177 for the combined model. Thus, although the FTIR-only model showed the highest ROC-AUC, the clinical-only model showed the best overall probability accuracy in this donor-blinded analysis, and the combined model was intermediate rather than superior on this metric. Recalibration parameters showed negative calibration intercepts (−0.668 to −1.076) and calibration slopes < 1 (0.279–0.528), indicating predicted probabilities that were overly extreme relative to observed event rates under strict donor-blinded validation (Figure 8). Such patterns are consistent with overfitting/over-dispersion in small-sample model development and support recalibration prior to clinical use [54,55].

#### 3.5.2. Precision–Recall Performance (PR-AUC)

PR-AUC was additionally evaluated to account for outcome imbalance in the FTIR-based analyses. In the primary QC-passed cohort (prevalence = 0.23), precision–recall characteristics were summarized using average precision computed from pooled out-of-fold predicted probabilities under donor-blinded grouped cross-validation. The baseline PR-AUC, corresponding to a non-informative classifier, was therefore 0.23. The combined clinical + FTIR model achieved a PR-AUC (average precision) of approximately 0.64, substantially exceeding the baseline prevalence and indicating meaningful enrichment of DGF-positive predictions beyond chance. These findings complement ROC-AUC-based discrimination metrics, but they should be interpreted alongside the donor-blinded ROC-AUC and Brier results, which did not show a clear overall advantage of the combined model over the clinical-only benchmark in this cohort.

#### 3.5.3. Clinical Usefulness (Donor-Blinded; Decision Curve Analysis)

**Figure 9:**
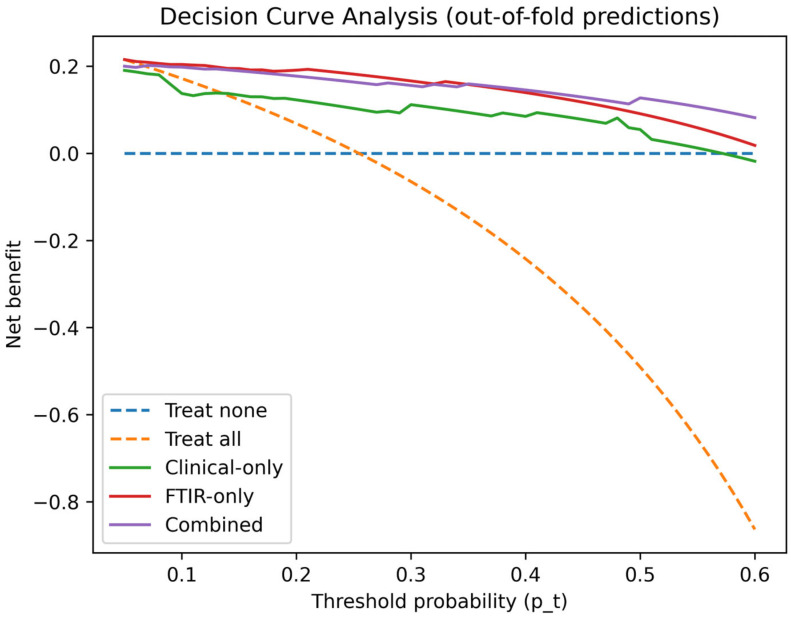
Decision Curve Analysis for DGF prediction models using donor-grouped out-of-fold probabilities. Decision curves show net benefit across threshold probabilities for clinical-only, FTIR-only, and combined models compared with treat-all and treat-none strategies. Probabilities were generated using donor-grouped StratifiedGroupKFold (5-fold).

Clinical usefulness was assessed with DCA, which summarizes net benefit across a range of threshold probabilities relative to “treat-all” and “treat-none” strategies [35]. Under donor-grouped validation, the combined model provided positive net benefit across clinically plausible thresholds; e.g., net benefit of 0.155 at p_t_ = 0.20 and 0.130 at p_t_ = 0.30. The clinical-only model showed similar performance at moderate thresholds (net benefit 0.114 at p_t_ = 0.20; 0.088 at p_t_ = 0.30). FTIR-only net benefit was higher at low thresholds (e.g., 0.194 at p_t_ = 0.10; 0.164 at p_t_ = 0.20) but declined at higher thresholds (net benefit 0.079 at p_t_ = 0.40 and 0.000 at p_t_ = 0.50), suggesting limited utility for high-certainty decision thresholds (Figure 9). These decision-analytic findings should nevertheless be interpreted in light of the suboptimal calibration observed under donor-blinded validation.

#### 3.5.4. “Plus” Analysis: Non-Blinded Validation to Illustrate Optimistic Bias

For transparency, we also repeated the same calibration and DCA analyses using standard stratified 5-fold cross-validation at the sample level (i.e., without grouping by donor). As expected, this approach yielded more optimistic performance, particularly for models incorporating spectral features. In the non-blinded setting, AUC increased to 0.840 for FTIR-only and 0.854 for the combined model (vs 0.814 and 0.796 under donor-blinded validation), and Brier scores improved to 0.143 (FTIR-only) and 0.129 (combined) (vs 0.194 and 0.177 when donor-blinded). DCA similarly showed larger net benefit estimates across thresholds (e.g., combined net benefit 0.177 at p_t_ = 0.20 and 0.156 at p_t_ = 0.30). These differences are consistent with performance inflation when correlated observations from the same donor can appear across folds, and therefore the donor-blinded results should be regarded as the primary internal validation.

Because the dataset includes paired kidneys from the same donor, we repeated calibration and decision curve analyses using donor-grouped cross-validation (Donor Code) to prevent data leakage across folds. Consistent with clustered data guidance, donor-grouped validation yielded more conservative calibration and net benefit estimates than standard sample-level cross-validation, which we report as an additional transparency analysis. Subgroup analyses (e.g., donor type and perfusion solution) were considered exploratory when strata were very small, as performance estimation can be unstable or undefined when one outcome class is absent in a test fold. Taken together, donor-blinded calibration and DCA support cautious clinical utility of probability-based decision support, while highlighting the need for external validation and model recalibration in independent cohorts.

## 4. Discussion

DGF remains one of the most clinically meaningful early complications after deceased donor kidney transplantation because it is a practical surrogate for peri-transplant acute kidney injury driven largely by IRI. Across cohorts and eras, DGF has been associated with higher rates of acute rejection and inferior graft outcomes, although effect sizes vary with donor quality, recipient risk, and how DGF is defined and managed [3,5,9]. A persistent challenge for prediction studies is that DGF is most often defined as dialysis requirement within the first 7 days, a definition that is pragmatic but vulnerable to center-level practice variation (e.g., thresholds for dialysis initiation, volume management, and clinician preference) [1,2]. This variability makes calibration and conservative validation particularly important when proposing probability-based clinical decision support tools.

The aim of this study was not to “explain” DGF mechanistically but to test a translationally useful premise: does the biochemical content captured in preservation fluid during static cold storage carry enough information to stratify DGF risk, and does it add to routine clinical predictors? The idea is grounded in biology: during cold ischemia and subsequent reperfusion, injured renal tissue can release proteins, metabolites, and inflammatory mediators into the surrounding preservation solution; therefore, the solution can function as a preimplantation “liquid biopsy” of graft stress. This concept has already been explored with targeted biomarker measurements in preservation fluid (e.g., inflammatory mediators), supporting the plausibility that cold storage fluids contain prognostic information about early graft dysfunction [56,57].

### 4.1. Benchmarking Our Clinical Results Against Prior Work

In our donor-clustered evaluation (treating donor ID as the clustering unit), the clinical-only model achieved moderate discrimination (AUC ≈ 0.78). This magnitude is consistent with the broader DGF prediction literature, where clinical models built from donor/recipient/transplant variables often achieve moderate c-statistics, but performance can vary and tends to soften in more rigorous validations or external cohorts [2,3,5,58,59]. This is also in line with the fact that DGF risk is shaped by a heterogeneous mix of pathways (ischemic injury, endothelial dysfunction, inflammatory activation, nephron mass constraints), which are only partially captured by routine clinical descriptors [3,60].

Importantly, our work sits in a setting where paired kidneys from a single donor can both enter the cohort. Large registry analyses have highlighted that donor quality and DGF interact with long-term outcomes and that risk factors such as KDPI and cold ischemia time are consistently relevant at the population level [60]. These observations strengthen the rationale for donor-aware modeling and validation in smaller mechanistic or biomarker-enriched cohorts such as ours, because donor biology is a meaningful shared source of correlation between paired kidneys.

### 4.2. Preservation Fluid Biomarkers: Where Our FTIR Spectroscopy Approach Fits

Targeted preservation fluid biomarker studies during static cold storage support the general principle that injury-related signals are measurable before implantation. For example, Baboudjian and colleagues reported that inflammatory markers measured in kidney preservation fluid during cold storage were associated with early graft dysfunction, illustrating that the cold storage environment contains clinically relevant inflammatory information [56]. Hall and colleagues similarly discuss preservation fluid as a biomarker source for DGF prediction and summarize evidence that cold storage effluent contains measurable injury signals [57]. At the same time, the broader biomarker literature also emphasizes that single analytes rarely provide consistently high accuracy across donor types, centers, and protocols; heterogeneity in procurement, storage duration, and recipient factors tends to dilute single-marker generalizability [57,61].

Our FTIR spectroscopy-based strategy addresses that limitation from the opposite direction: rather than selecting one or two biomarkers a priori, FTIR provides a rapid, label-free biochemical “fingerprint” across the mid-infrared region [22,24,25]. In donor-clustered validation, the FTIR-only model achieved AUC ≈ 0.81, placing it in a competitive range compared with many targeted approaches, while remaining operationally much lighter than metabolomics platforms. More complex omics studies of preservation/perfusion fluids (e.g., metabolomics) also support the notion that early dysfunction correlates with multifeature biochemical patterns rather than with a single dominant marker; FTIR spectroscopy based platform can be viewed as a practical, lower-cost entry point into that multivariate signal space, although with less molecular specificity [57,61,62,63].

### 4.3. Why Preservation Solution Matters and Why We Should Not Ignore It

One of the most important practical findings in our analysis is that preservation solution chemistry can dominate spectral variance. This is expected: solutions such as Celsior^®^ and Custodiol^®^/HTK differ substantially in buffer systems, osmotic agents, and antioxidant components, and FTIR spectroscopy is intrinsically sensitive to such compositional differences. In the kidney preservation literature, evidence suggests that commonly used static cold storage solutions (UW, HTK, Celsior^®^) often yield broadly similar clinical outcomes in many settings, and randomized or comparative data frequently show no large differences in DGF risk between these solutions, though context-dependent nuances exist [26,64,65].

That external evidence guides how we should interpret solution effects in our study. If different solutions tend to have similar average clinical outcomes, then solution “signatures” in FTIR spectra are not automatically causal signals for DGF; instead, they can act as background chemistry and a potential confounder if solution choice correlates with donor type, procurement pathway, or ischemia exposure. For that reason, we believe future translational work should pre-specify strategies for solution handling (e.g., solution-specific models; inclusion of solution as a stratification variable; or subtraction/normalization against matched blank solution references). This is a practical design issue for generalization, not a minor technicality.

### 4.4. Combined Modeling: Complementarity Is Plausible, but Not Guaranteed in Small Cohorts

A common expectation is that “clinical + spectroscopy” must outperform either alone. In our donor-clustered evaluation, the combined model achieved AUC ≈ 0.80, which did not uniformly exceed the FTIR spectroscopy-only model (≈0.81). This is not surprising in a modest dataset, as combined models can fail to dominate when (i) the additional modality contributes overlapping information or (ii) sample size is insufficient to learn complementary structure without overfitting. The fact that FTIR-only remained competitive under donor-clustered validation is encouraging, because it suggests preservation fluid contains meaningful signal not trivially reducible to clinical variables; however, larger cohorts will be needed to determine whether combined modeling consistently improves performance across donor types and solution strata.

### 4.5. Why Donor-Clustered (“Donor-Blinded”) Validation Is the Right Primary Analysis

Because our dataset includes paired kidneys, we explicitly evaluated models under two regimes: standard sample-level cross-validation and donor-clustered cross-validation. The difference matters—in clustered data settings, leakage occurs when correlated observations from the same donor appear in both training and testing folds; this can inflate discrimination and calibration because donor-specific biology and procurement context effectively “leak” into the test fold. TRIPOD-Cluster was created specifically to improve transparency in prediction modeling using clustered data, reflecting how common and consequential this issue is [32,66].

In this study, the donor-clustered evaluation produced more conservative discrimination and less favorable calibration than standard cross-validation, which is consistent with what prediction methodology papers describe as optimism under less strict internal validation. That observation is not a weakness; it is a credibility feature. It tells reviewers we are not overstating performance and that we understand the data structure intrinsic to deceased donor transplantation research.

### 4.6. Calibration and Clinical Usefulness: Translating AUC into Decisions

Another reviewer-critical point is that transplant clinicians do not act on AUC; they act on risk thresholds. Because DGF prediction is intended to guide early care intensity rather than “diagnose” a pathologic entity, calibration and decision utility are central. DGF’s clinical definition is itself practice-dependent, so a model that ranks risk well but outputs miscalibrated probabilities can still mislead threshold-based decisions [1,2].

Decision curve analysis (DCA) provides a pragmatic bridge between prediction and action by estimating net benefit across threshold probabilities compared with “treat-all” and “treat-none” strategies. Under donor-clustered validation, our DCA suggested that the combined model provides positive net benefit across clinically plausible thresholds, while FTIR-only may be particularly helpful at lower thresholds where sensitivity-leaning triage is preferred. This supports a realistic framing: FTIR-based risk could help programs to identify kidneys warranting intensified early surveillance, proactive dialysis logistics, or closer attention to hemodynamic and immunosuppression factors without claiming that FTIR is a mechanistic assay of IRI. Accordingly, although DCA suggests potential clinical usefulness, these models should not be used for probability-based clinical decision making without external validation and recalibration in independent cohorts. In practical terms, the current models should be viewed as ranking tools under development, not as clinically reliable probability estimators.

### 4.7. Limitations and Next Steps

This study has limitations that should be acknowledged directly. First, it is a single-center cohort with limited sample size, and subgroup analyses by donor type and preservation solution become statistically fragile when events are sparse; this explains why some stratified conditions yield unstable or undefined metrics and should be interpreted as exploratory rather than definitive. Second, individual cold ischemia time and warm ischemia time were not retrievable in this cohort and therefore could not be included as candidate predictors. Because ischemia exposure is a major determinant of DGF risk, we handled this limitation analytically by retaining donor pathway (DBD vs. DCD) as a structured proxy, but this does not fully replace case-level ischemia timing data. Third, DGF definition variability across centers can influence apparent performance and calibration, making external validation essential before any clinical deployment [1,2].

Fourth, FTIR is integrative but not molecule-specific; spectral “features” should not be over-interpreted as specific biochemical entities without orthogonal validation. Finally, while donor-clustered validation reduces optimistic bias, it does not replace external validation across different procurement systems, solution usage patterns, and dialysis thresholds. Future translational work would also benefit from prospective multicenter designs with harmonized data collection and, where feasible, multimodal validation against complementary biochemical, perfusion, or histologic readouts.

Despite these constraints, our findings support a more modest conclusion: static cold storage preservation fluid contains measurable biochemical signal that can be captured rapidly by FTIR and used for exploratory DGF risk stratification. In the donor-blinded analysis, the FTIR-only model showed the highest ROC-AUC, but combining FTIR with clinical variables did not improve discrimination over the clinical-only model and did not yield the best Brier score. Accordingly, the principal contribution of this study is proof of feasibility and a leakage-aware methodological framework for future external validation and model updating in larger cohorts. Future work should prioritize multicenter prospective cohorts with harmonized sampling, explicit solution handling strategies, donor-clustered validation plans, and external recalibration steps that align directly with clustered prediction model guidance [32,66].

Future prospective studies should also incorporate more granular endpoint definitions, including dialysis indication, number of dialysis sessions, DGF duration, and composite early graft dysfunction endpoints. In addition, larger studies should compare donor-grouped nested validation schemes, stronger penalization strategies, and more stable dimensionality reduction approaches.

## 5. Conclusions

In this study of 56 deceased donor kidney transplants (25% DGF), FTIR spectra obtained from static cold storage preservation fluid contained measurable biochemical information associated with DGF. Donor-blinded validation, in which paired kidneys from the same donor were kept within the same fold, was essential to reduce optimistic bias from within-donor correlation.

In the primary donor-blinded analysis, the FTIR-only model showed the highest ROC-AUC (0.814), the clinical-only model showed the lowest Brier score (0.162), and the combined model showed intermediate ROC-AUC (0.796) and Brier score (0.177). Thus, in this cohort, combining FTIR with clinical variables did not demonstrate a clear incremental improvement in discrimination over the clinical-only benchmark. All performance estimates, particularly subgroup benchmark estimates, should be regarded as exploratory and potentially unstable because of the small sample size and sparse events. These results support FTIR preservation fluid profiling as a promising proof-of-concept adjunct for future model development, but not yet as a clinically deployable combined predictor. The next steps are external validation in larger multicenter cohorts, standardized pre-analytical handling and QC, and model updating/recalibration to obtain reliable probability estimates.

## Figures and Tables

**Table 1:** Baseline characteristics by delayed graft function.

Variable	Overall (*n* = 56)	DGF No (*n* = 42)	DGF Yes (*n* = 14)	*p* Value
Donor age, years	48.54 ± 17.24	47.43 ± 18.42	51.86 ± 13.08	0.333 ^a^
Donor weight, kg	70.00 [62.00, 75.00] (*n* = 54)	70.00 [60.00, 75.00] (*n* = 42)	73.00 [70.00, 75.00] (*n* = 14)	0.459 ^b^
Donor height, cm	166.50 [160.00, 175.00] (*n* = 54)	165.00 [160.00, 175.00] (*n* = 42)	172.00 [165.00, 175.00] (*n* = 14)	0.334 ^b^
Donor BMI, kg/m^2^	25.14 ± 3.78 (*n* = 54)	25.19 ± 3.89 (*n* = 42)	25.00 ± 3.56 (*n* = 14)	0.874 ^a^
Donor serum creatinine, mg/dL	0.95 [0.67, 1.11]	0.80 [0.60, 1.05]	1.08 [0.97, 1.47]	0.008 ^b^
Donor urea, mg/dL	32.00 [21.00, 41.00]	32.00 [21.00, 41.00]	33.00 [22.75, 38.25]	0.880 ^b^
Donor eGFR, mL/min/1.73 m^2^	85.59 ± 29.72	90.86 ± 29.73	69.79 ± 24.32	0.013 ^a^
KDPI, %	51.17 ± 26.54 (*n* = 54)	46.93 ± 25.81 (*n* = 41) *	64.54 ± 25.23 (*n* = 13) *	0.041 ^a^
KDRI	1.04 ± 0.28 (*n* = 54)	0.99 ± 0.26 (*n* = 41) *	1.20 ± 0.31 (*n* = 13) *	0.044 ^a^
Recipient age at transplant, years	49.00 [39.00, 63.00]	47.00 [38.00, 62.25]	58.00 [51.25, 65.25]	0.046 ^b^
Time on renal replacement therapy, days	1717.50 [1168.50, 2505.00]	1732.50 [848.75, 2449.75]	1661.50 [1473.50, 2555.00]	0.755 ^b^
Total HLA mismatches (A, B, C, DR)	6.00 [4.00, 7.00]	6.00 [4.00, 7.00]	5.50 [3.00, 6.75]	0.360 ^b^
HLA-A mismatches	1.00 [1.00, 2.00]	1.00 [1.00, 2.00]	1.00 [1.00, 2.00]	0.974 ^b^
HLA-B mismatches	2.00 [1.00, 2.00]	2.00 [1.00, 2.00]	2.00 [0.25, 2.00]	0.629 ^b^
HLA-C mismatches	1.00 [1.00, 2.00]	2.00 [1.00, 2.00]	1.00 [1.00, 1.75]	0.211 ^b^
HLA-DR mismatches	1.00 [1.00, 2.00]	1.00 [1.00, 2.00]	1.00 [0.25, 2.00]	0.249 ^b^
Donor sex, male	28 (50.0%)	19 (45.2%)	9 (64.3%)	0.217 ^c^
Donor hypertension, yes	21 (37.5%)	15 (35.7%)	6 (42.9%)	0.633 ^c^
Donor diabetes, yes	4 (7.1%)	2 (4.8%)	2 (14.3%)	0.258 ^d^
Donor Smoking, yes	9 (16.4%)	7 (16.7%)	2 (15.4%)	1.000 ^d^
Donor type, DCD	13 (23.2%)	3 (7.1%)	10 (71.4%)	<0.001 ^d^
Donor ethnicity				0.383 ^c^
African	2 (3.6%)	2 (4.8%)	0 (0.0%)	
White	47 (83.9%)	36 (85.7%)	11 (78.6%)	
Unknown	7 (12.5%)	4 (9.5%)	3 (21.4%)	
Donor cause of death				<0.001 ^c^
CRA	13 (23.2%)	3 (7.1%)	10 (71.4%)	
CVA	25 (44.6%)	23 (54.8%)	2 (14.3%)	
HYPOXIA	5 (8.9%)	5 (11.9%)	0 (0.0%)	
TBI	13 (23.2%)	11 (26.2%)	2 (14.3%)	
Perfusion solution, Celsior^®^	48 (85.7%)	36 (85.7%)	12 (85.7%)	1.000 ^d^
Donor cardiorespiratory arrest, yes	25 (44.6%)	15 (35.7%)	10 (71.4%)	0.020 ^c^
Recipient sex, male	39 (69.6%)	29 (69.0%)	10 (71.4%)	1.000 ^d^
Recipient ethnicity				0.050 ^c^
African	13 (23.2%)	6 (14.3%)	7 (50.0%)	
Asian	1 (1.8%)	1 (2.4%)	0 (0.0%)	
White	41 (73.2%)	34 (81.0%)	7 (50.0%)	
Mixed	1 (1.8%)	1 (2.4%)	0 (0.0%)	
Previous transplant, any	8 (14.3%)	6 (14.3%)	2 (14.3%)	1.000 ^d^
Renal replacement therapy modality				0.843 ^c^
Hemodialysis	47 (83.9%)	35 (83.3%)	12 (85.7%)	
Peritoneal dialysis	6 (10.7%)	5 (11.9%)	1 (7.1%)	
Preemptive	3 (5.4%)	2 (4.8%)	1 (7.1%)	

^a^ Welch’s *t*-test; ^b^ Mann–Whitney U; ^c^ Chi-square; ^d^ Fisher’s exact; * missing data. eGFR, estimated glomerular filtration rate; KDPI, Kidney Donor Profile Index; KDRI, Kidney Donor Risk Index; DCD, Donation after Circulatory Death; CRA, Cardiorespiratory Arrest; CVA, Cerebrovascular Accident TBI, Traumatic Brain Injury. Continuous variables were summarized as mean ± SD when approximately normally distributed and as median [IQR] otherwise; normality was assessed using the Shapiro–Wilk test for small samples. Group comparisons used Welch’s *t*-test or Mann–Whitney U as appropriate, and categorical variables used chi-square or Fisher’s exact test depending on expected cell counts.

**Table 2:** Group comparison of FTIR spectral quality control metrics by delayed graft function status. Values are reported as median [interquartile range (IQR)]. Between-group comparisons used two-sided Mann–Whitney U tests; effect size is reported as the rank-biserial correlation (r_rb; range −1 to +1, derived from U and sample sizes).

Metric	DGF_yes Median [IQR]	DGF_no Median [IQR]	U	*p*	rank_biserial_r	DGF_yes Mean ± SD	DGF_no Mean ± SD
SNR_AmideI	68.17 [61.97,129.49]	74.03 [59.85,83.85]	310.50	0.76	−0.06	95.94 ± 70.36	77.94 ± 53.61
Spike_count	16 [8.45,75]	11.5 [5.25,26.5]	346.00	0.33	−0.18	24.79 ± 21.02	18.48 ± 17.28
Cosine_fp	0.995 [0.89,0.997]	0.994 [0.95,0.997]	279.50	0.79	0.05	0.94 ± 0.07	0.956 ± 0.11
Baseline_frac	0.197 [0.143,0.23]	0.21 [0.15,0.25]	267.50	0.62	0.09	0.22 ± 0.18	0.85 ± 3.92

DGF, delayed graft function; FTIR, Fourier transform infrared; SNR, signal-to-noise ratio; IQR, interquartile range.

## Data Availability

The de-identified study data are not publicly available because of institutional governance and patient-confidentiality restrictions. Access may be considered by the corresponding author for academic verification purposes, subject to approval by the host institution and applicable data-protection requirements.

## Supplementary Materials

The following supporting information can be downloaded at: https://www.mdpi.com/article/10.3390/jcm15072762/s1, Supplementary File S1: Completed TRIPOD+AI checklist. Supplementary Table S1: FTIR spectral quality control metrics. Supplementary Table S2: Exploratory benchmark performance of clinical-only models. Supplementary Table S3: Ranked clinical predictors selected by FCBF. Supplementary Table S4: Exploratory benchmark performance of FTIR-only models. Supplementary Table S5: Exploratory benchmark performance of combined clinical + FTIR models.

## Abbreviations

The following abbreviations are used in this manuscript:

IRI	Ischemia–reperfusion injury
DGF	Delayed graft function
CIT	Cold ischemia time
FTIR	Fourier transform infrared spectroscopy
AI	Artificial intelligence
ML	Machine learning
DCA	Decision curve analysis
ULS São José	Unidade Local de Saúde de São José
WIT	Warm ischemia time
QC	Quality control
SNR	Signal-to-noise ratio
MDS	Multidimensional scaling
HCA	Ward hierarchical clustering
t-SNE	t-distributed stochastic neighbor embedding
FCBF	Fast correlation-based filter
KDPI	Kidney Donor Profile Index
KDRI	Kidney Donor Risk Index
PR-AUC	Area under the precision–recall curve
ROC-AUC	Area under the receiver operating characteristic curve
CRA	Cardiorespiratory Arrest
CVA	Cerebrovascular Accident
TBI	Traumatic Brain Injury
PCA	Principal component analysis
CV	Cross validation

## References

[1] Yarlagadda S.G., Coca S.G., Garg A.X., Doshi M., Poggio E., Marcus R.J., Parikh C.R. (2008). Marked Variation in the Definition and Diagnosis of Delayed Graft Function: A Systematic Review. Nephrol. Dial. Transplant..

[2] Orandi B.J., James N.T., Hall E.C., Van Arendonk K.J., Garonzik-Wang J.M., Gupta N., Montgomery R.A., Desai N.M., Segev D.L. (2015). Center-Level Variation in the Development of Delayed Graft Function After Deceased Donor Kidney Transplantation. Transplantation.

[3] Siedlecki A., Irish W., Brennan D.C. (2011). Delayed Graft Function in the Kidney Transplant. Am. J. Transplant..

[4] Tapiawala S.N., Tinckam K.J., Cardella C.J., Schiff J., Cattran D.C., Cole E.H., Kim S.J. (2010). Delayed Graft Function and the Risk for Death with a Functioning Graft. J. Am. Soc. Nephrol. JASN.

[5] Li M.T., Ramakrishnan A., Yu M., Daniel E., Sandra V., Sanichar N., King K.L., Stevens J.S., Husain S.A., Mohan S. (2023). Effects of Delayed Graft Function on Transplant Outcomes: A Meta-Analysis. Transplant. Direct.

[6] Budhiraja P., Reddy K.S., Butterfield R.J., Jadlowiec C.C., Moss A.A., Khamash H.A., Kodali L., Misra S.S., Heilman R.L. (2022). Duration of Delayed Graft Function and Its Impact on Graft Outcomes in Deceased Donor Kidney Transplantation. BMC Nephrol..

[7] Warmuzińska N., Łuczykowski K., Stryjak I., Wojtal E., Woderska-Jasińska A., Masztalerz M., Włodarczyk Z., Bojko B. (2024). Metabolomic and Lipidomic Profiling for Pre-Transplant Assessment of Delayed Graft Function Risk Using Chemical Biopsy with Microextraction Probes. Int. J. Mol. Sci..

[8] Nieuwenhuijs-Moeke G.J., Pischke S.E., Berger S.P., Sanders J.S.F., Pol R.A., Struys M.M.R.F., Ploeg R.J., Leuvenink H.G.D. (2020). Ischemia and Reperfusion Injury in Kidney Transplantation: Relevant Mechanisms in Injury and Repair. J. Clin. Med..

[9] Wu W.K., Famure O., Li Y., Kim S.J. (2015). Delayed Graft Function and the Risk of Acute Rejection in the Modern Era of Kidney Transplantation. Kidney Int..

[10] Perico N., Cattaneo D., Sayegh M.H., Remuzzi G. (2004). Delayed Graft Function in Kidney Transplantation. Lancet.

[11] Ahlmark A., Sallinen V., Räisänen-Sokolowski A., Ahopelto K., Lempinen M., Lauronen J., Helanterä I. (2025). Protocol Biopsies in Delayed Graft Function Kidney Transplants From Brain-Dead Donors. Clin. Transplant..

[12] Troppmann C., Gillingham K.J., Benedetti E., Almond P.S., Gruessner R.W., Najarian J.S., Matas A.J. (1995). Delayed Graft Function, Acute Rejection, and Outcome after Cadaver Renal Transplantation. The Multivariate Analysis. Transplantation.

[13] Motter J.D., Jackson K.R., Long J.J., Waldram M.M., Orandi B.J., Montgomery R.A., Stegall M.D., Jordan S.C., Benedetti E., Dunn T.B. (2021). Delayed Graft Function and Acute Rejection Following HLA-Incompatible Living Donor Kidney Transplantation. Am. J. Transplant. Off. J. Am. Soc. Transplant. Am. Soc. Transpl. Surg..

[14] Debout A., Foucher Y., Trébern-Launay K., Legendre C., Kreis H., Mourad G., Garrigue V., Morelon E., Buron F., Rostaing L. (2015). Each Additional Hour of Cold Ischemia Time Significantly Increases the Risk of Graft Failure and Mortality Following Renal Transplantation. Kidney Int..

[15] Moers C., Smits J.M., Maathuis M.-H.J., Treckmann J., van Gelder F., Napieralski B.P., van Kasterop-Kutz M., van der Heide J.J.H., Squifflet J.-P., van Heurn E. (2009). Machine Perfusion or Cold Storage in Deceased-Donor Kidney Transplantation. N. Engl. J. Med..

[16] Jochmans I., Brat A., Davies L., Hofker H.S., van de Leemkolk F.E.M., Leuvenink H.G.D., Knight S.R., Pirenne J., Ploeg R.J., Abramowicz D. (2020). Oxygenated versus Standard Cold Perfusion Preservation in Kidney Transplantation (COMPARE): A Randomised, Double-Blind, Paired, Phase 3 Trial. Lancet.

[17] Hosgood S.A., Callaghan C.J., Wilson C.H., Smith L., Mullings J., Mehew J., Oniscu G.C., Phillips B.L., Bates L., Nicholson M.L. (2023). Normothermic Machine Perfusion versus Static Cold Storage in Donation after Circulatory Death Kidney Transplantation: A Randomized Controlled Trial. Nat. Med..

[18] Guzzi F., Knight S.R., Ploeg R.J., Hunter J.P. (2020). A Systematic Review to Identify Whether Perfusate Biomarkers Produced during Hypothermic Machine Perfusion Can Predict Graft Outcomes in Kidney Transplantation. Transpl. Int..

[19] Crocetto F., Amicuzi U., Musone M., Magliocchetti M., Di Lieto D., Tammaro S., Pastore A.L., Fuschi A., Falabella R., Ferro M. (2025). Liquid Biopsy: Current Advancements in Clinical Practice for Bladder Cancer. J. Liq. Biopsy.

[20] Pisapia P., Pepe F., Russo G., Capoluongo R., Coppola M., Giudice F.D., Ferro M., Madonna A., Musone M., Troncone G. (2025). Liquid Biopsy Testing in Urological Cancers: Focus on Urine. Urol. Oncol..

[21] Liu R.X., Koyawala N., Thiessen-Philbrook H.R., Doshi M.D., Reese P.P., Hall I.E., Mohan S., Parikh C.R. (2023). Untargeted Metabolomics of Perfusate and Their Association with Hypothermic Machine Perfusion and Allograft Failure. Kidney Int..

[22] Ramalhete L., Araújo R., Vieira M.B., Vigia E., Pena A., Carrelha S., Ferreira A., Calado C.R.C. (2025). Rapid FTIR Spectral Fingerprinting of Kidney Allograft Perfusion Fluids Distinguishes DCD from DBD Donors: A Pilot Machine Learning Study. Metabolites.

[23] Baker M.J., Trevisan J., Bassan P., Bhargava R., Butler H.J., Dorling K.M., Fielden P.R., Fogarty S.W., Fullwood N.J., Heys K.A. (2014). Using Fourier Transform IR Spectroscopy to Analyze Biological Materials. Nat. Protoc..

[24] Ramalhete L., Araújo R., Vieira M.B., Vigia E., Aires I., Ferreira A., Calado C.R.C. (2025). Integration of FTIR Spectroscopy and Machine Learning for Kidney Allograft Rejection: A Complementary Diagnostic Tool. J. Clin. Med..

[25] Ramalhete L., Vieira M.B., Araújo R., Vigia E., Aires I., Ferreira A., Calado C.R.C. (2024). Predicting Cellular Rejection of Renal Allograft Based on the Serum Proteomic Fingerprint. Int. J. Mol. Sci..

[26] O’Callaghan J.M., Knight S.R., Morgan R.D., Morris P.J. (2012). Preservation Solutions for Static Cold Storage of Kidney Allografts: A Systematic Review and Meta-Analysis. Am. J. Transplant..

[27] Faenza A., Catena F., Nardo B., Montalti R., Capocasale E., Busi N., Boggi U., Vistoli F., Di Naro A., Albertazzi A. (2001). Kidney Preservation with University of Wisconsin and Celsior Solution: A Prospective Multicenter Randomized Study. Transplantation.

[28] Ackemann J., Gross W., Mory M., Schaefer M., Gebhard M.-M. (2002). Celsior versus Custodiol: Early Postischemic Recovery after Cardioplegia and Ischemia at 5 Degrees C. Ann. Thorac. Surg..

[29] Zuluaga G.L.L., Agudelo R.E.S., Tobón J.J.Z. (2013). Preservation Solutions for Liver Transplantation in Adults: Celsior versus Custodiol: A Systematic Review and Meta-Analysis with an Indirect Comparison of Randomized Trials. Transplant. Proc..

[30] Rijkse E., Ceuppens S., Qi H., IJzermans J.N.M., Hesselink D.A., Minnee R.C. (2021). Implementation of Donation after Circulatory Death Kidney Transplantation Can Safely Enlarge the Donor Pool: A Systematic Review and Meta-Analysis. Int. J. Surg..

[31] Collins G.S., Reitsma J.B., Altman D.G., Moons K.G.M. (2015). Transparent Reporting of a Multivariable Prediction Model for Individual Prognosis or Diagnosis (TRIPOD): The TRIPOD Statement. Ann. Intern. Med..

[32] Collins G.S., Moons K.G.M., Dhiman P., Riley R.D., Beam A.L., Van Calster B., Ghassemi M., Liu X., Reitsma J.B., van Smeden M. (2024). TRIPOD+AI Statement: Updated Guidance for Reporting Clinical Prediction Models That Use Regression or Machine Learning Methods. BMJ.

[33] Wolff R.F., Moons K.G.M., Riley R.D., Whiting P.F., Westwood M., Collins G.S., Reitsma J.B., Kleijnen J., Mallett S. (2019). PROBAST: A Tool to Assess the Risk of Bias and Applicability of Prediction Model Studies. Ann. Intern. Med..

[34] Moons K.G.M., Damen J.A.A., Kaul T., Hooft L., Andaur Navarro C., Dhiman P., Beam A.L., Van Calster B., Celi L.A., Denaxas S. (2025). PROBAST+AI: An Updated Quality, Risk of Bias, and Applicability Assessment Tool for Prediction Models Using Regression or Artificial Intelligence Methods. BMJ (Clin. Res. Ed.).

[35] Vickers A.J., Elkin E.B. (2006). Decision Curve Analysis: A Novel Method for Evaluating Prediction Models. Med. Decis. Mak. Int. J. Soc. Med. Decis. Mak..

[36] Vickers A.J., Van Calster B., Steyerberg E.W. (2016). Net Benefit Approaches to the Evaluation of Prediction Models, Molecular Markers, and Diagnostic Tests. BMJ.

[37] Van Calster B., Wynants L., Verbeek J.F.M., Verbakel J.Y., Christodoulou E., Vickers A.J., Roobol M.J., Steyerberg E.W. (2018). Reporting and Interpreting Decision Curve Analysis: A Guide for Investigators. Eur. Urol..

[38] Manara A.R., Murphy P.G., O’Callaghan G. (2012). Donation after Circulatory Death. Br. J. Anaesth..

[39] Burgess M., Silsby L., Madden S., Letra J.D.S., Thomson S., Tate J., Mitchinson S., Hurley K., Hurley R., Bon M. (2025). Eligible DBD Donors Proceeding via the DCD Pathway: Incidence, Cause, and Outcomes in the United Kingdom. Transplant. Direct.

[40] Kasiske B.L., Zeier M.G., Chapman J.R., Craig J.C., Ekberg H., Garvey C.A., Green M.D., Jha V., Josephson M.A., Kiberd B.A. (2010). KDIGO Clinical Practice Guideline for the Care of Kidney Transplant Recipients: A Summary. Kidney Int..

[41] González-Domínguez R., González-Domínguez Á., Sayago A., Fernández-Recamales Á. (2020). Recommendations and Best Practices for Standardizing the Pre-Analytical Processing of Blood and Urine Samples in Metabolomics. Metabolites.

[42] Buchanan J.L., Tormes Vaquerano J., Taylor E.B. (2022). Isolated Effects of Plasma Freezing versus Thawing on Metabolite Stability. Metabolites.

[43] Araújo R., Ramalhete L., Ribeiro E., Calado C. (2022). Plasma versus Serum Analysis by FTIR Spectroscopy to Capture the Human Physiological State. BioTech.

[44] Mariani M., He S., McHugh M., Andreoli M., Pandya D., Sieber S., Wu Z., Fiedler P., Shahabi S., Ferlini C. (2014). Integrated Multidimensional Analysis Is Required for Accurate Prognostic Biomarkers in Colorectal Cancer. PLoS ONE.

[45] Liu Z., Ma R., Zhong Y. (2025). Assessing and Improving Reliability of Neighbor Embedding Methods: A Map-Continuity Perspective. Nat. Commun..

[46] Irish W.D., Ilsley J.N., Schnitzler M.A., Feng S., Brennan D.C. (2010). A Risk Prediction Model for Delayed Graft Function in the Current Era of Deceased Donor Renal Transplantation. Am. J. Transplant..

[47] Yu L., Liu H. (2003). Feature Selection for High-Dimensional Data: A Fast Correlation-Based Filter Solution. Proceedings of the Twentieth International Conference on Machine Learning Volume.

[48] Varma S., Simon R. (2006). Bias in Error Estimation When Using Cross-Validation for Model Selection. BMC Bioinform..

[49] Saito T., Rehmsmeier M. (2015). The Precision-Recall Plot Is More Informative than the ROC Plot When Evaluating Binary Classifiers on Imbalanced Datasets. PLoS ONE.

[50] Pettit R.W., Marlatt B.B., Miles T.J., Uzgoren S., Corr S.J., Shetty A., Havelka J., Rana A. (2023). The Utility of Machine Learning for Predicting Donor Discard in Abdominal Transplantation. Clin. Transplant..

[51] Steyerberg E.W., Vickers A.J., Cook N.R., Gerds T., Gonen M., Obuchowski N., Pencina M.J., Kattan M.W. (2010). Assessing the Performance of Prediction Models: A Framework for Traditional and Novel Measures. Epidemiology.

[52] Theakstone A.G., Rinaldi C., Butler H.J., Cameron J.M., Confield L.R., Rutherford S.H., Sala A., Sangamnerkar S., Baker M.J. (2021). Fourier-Transform Infrared Spectroscopy of Biofluids: A Practical Approach. Transl. Biophotonics.

[53] Yan C. (2025). A Review on Spectral Data Preprocessing Techniques for Machine Learning and Quantitative Analysis. iScience.

[54] Van Calster B., McLernon D.J., van Smeden M., Wynants L., Steyerberg E.W. (2019). Calibration: The Achilles Heel of Predictive Analytics. BMC Med..

[55] Steyerberg E.W., Vergouwe Y. (2014). Towards Better Clinical Prediction Models: Seven Steps for Development and an ABCD for Validation. Eur. Heart J..

[56] Baboudjian M., Gondran-Tellier B., Boissier R., Ancel P., Marjollet J., Lyonnet L., François P., Sabatier F., Lechevallier E., Dutour A. (2022). An Enhanced Level of VCAM in Transplant Preservation Fluid Is an Independent Predictor of Early Kidney Allograft Dysfunction. Front. Immunol..

[57] Hall I.E. (2017). Can Preservation Fluid Biomarkers Predict Delayed Graft Function in Transplanted Kidneys?. Clin. J. Am. Soc. Nephrol. CJASN.

[58] Scurt F.G., Ernst A., FischerFröhlich C.-L., Schwarz A., Becker J.U., Chatzikyrkou C. (2023). Performance of Scores Predicting Adverse Outcomes in Procurement Kidney Biopsies From Deceased Donors with Organs of Lower-Than-Average Quality. Transpl. Int. Off. J. Eur. Soc. Organ Transplant..

[59] Chapal M., Le Borgne F., Legendre C., Kreis H., Mourad G., Garrigue V., Morelon E., Buron F., Rostaing L., Kamar N. (2014). A Useful Scoring System for the Prediction and Management of Delayed Graft Function Following Kidney Transplantation from Cadaveric Donors. Kidney Int..

[60] Ahlmark A., Sallinen V., Eerola V., Lempinen M., Helanterä I. (2024). Characteristics of Delayed Graft Function and Long-Term Outcomes After Kidney Transplantation from Brain-Dead Donors: A Single-Center and Multicenter Registry-Based Retrospective Study. Transpl. Int..

[61] Baryła M., Skrzycki M., Danielewicz R., Kosieradzki M., Struga M. (2024). Protein Biomarkers in Assessing Kidney Quality before Transplantation-current Status and Future Perspectives (Review). Int. J. Mol. Med..

[62] Ramalhete L.M., Araújo R., Ferreira A., Calado C.R.C. (2022). Proteomics for Biomarker Discovery for Diagnosis and Prognosis of Kidney Transplantation Rejection. Proteomes.

[63] Avramidou E., Srinivasan D., Todorov D., Tsoulfas G., Papalois V. (2024). Diagnostic and Prognostic Value of Machine Perfusion Biomarkers in Kidney Graft Evaluation. Transplant. Proc..

[64] Hosgood S.A., Brown R.J., Nicholson M.L. (2021). Advances in Kidney Preservation Techniques and Their Application in Clinical Practice. Transplantation.

[65] Chen Y., Shi J., Xia T.C., Xu R., He X., Xia Y. (2019). Preservation Solutions for Kidney Transplantation: History, Advances and Mechanisms. Cell Transplant..

[66] Debray T.P.A., Collins G.S., Riley R.D., Snell K.I.E., Van Calster B., Reitsma J.B., Moons K.G.M. (2023). Transparent Reporting of Multivariable Prediction Models Developed or Validated Using Clustered Data: TRIPOD-Cluster Checklist. BMJ (Clin. Res. Ed.).

