# Supplementary material for: Machine Learning-Assisted FTIR Spectroscopy Analysis of Kidney Preservation Fluids for Delayed Graft Function Risk Stratification
Source: J Clin Med. 2026 Apr 6;15(7):2762. doi: 10.3390/jcm15072762 (PMC13073485; doi:10.3390/jcm15072762)
Supplement: Supplementary file 1 [file jcm-15-02762-s001.zip › jcm-4201226-supplementary.pdf]

Supplementary Materials:

Supplementary File S1: Completed TRIPOD+AI checklist. Supplementary Table S1: FTIR spectral quality-control metrics. Supplementary Table S2: Exploratory benchmark performance of clinical-only models. Supplementary Table S3: Ranked clinical predictors selected by FCBF. Supplementary Table S4: Exploratory benchmark performance of FTIR-only models. Supplementary Table S5: Exploratory benchmark performance of combined clinical + FTIR models.

Supplementary File S1. Completed TRIPOD+AI Checklist

This checklist was completed against the current revised manuscript and the condensed official TRIPOD+AI checklist. Because page numbers can shift during revision and journal typesetting, the "Reported in manuscript" field uses manuscript section references rather than fixed page numbers.

TITLE / ABSTRACT / INTRODUCTION

Section/Topic	Item	D/E	Checklist item	Reported in manuscript
Title	1	D;E	Identify the study as a prediction-model study, target population, and outcome.	Title
Abstract	2	D;E	Provide a structured summary covering design, data, participants, predictors, outcome, methods, results, and conclusions.	Abstract
Background	3a	D;E	Explain the clinical context and rationale, including references to existing models.	Introduction
Background	3b	D;E	Describe the target population, intended purpose in the care pathway, and intended users.	Introduction; 4.6
Background	3c	D;E	Describe known health inequalities between sociodemographic groups.	Not applicable
Objectives	4	D;E	State whether the study concerns model development, evaluation, or both.	Abstract; end of Introduction

METHODS

Section/Topic	Item	D/E	Checklist item	Reported in manuscript
Data	5a	D;E	Describe data source(s), rationale, and representativeness.	2.1.1
Data	5b	D;E	Specify dates of participant accrual and, if applicable, end of follow-up.	Not applicable
Participants	6a	D;E	Describe study setting, number and location of centers.	2.1.1
Participants	6b	D;E	Describe eligibility criteria.	2.1.3
Participants	6c	D;E	Give details of treatments received and how handled, if relevant.	2.1.7; Table 1
Data preparation	7	D;E	Describe preprocessing and quality checking.	2.2.1–2.3.3; 2.5.2; 2.5.4; 3.2
Outcome	8a	D;E	Clearly define outcome, timing, assessment, and rationale.	2.1.7; Introduction

Section/Topic	Item	D/E	Checklist item	Reported in manuscript
Outcome	8b	D;E	If subjective outcome assessment was required, describe assessors.	2.1.7
Outcome	8c	D;E	Report blinding of outcome assessment to predictors.	2.1.1; 2.1.7
Predictors	9a	D	Describe choice of initial predictors and any pre-selection before model building.	2.1.6; 2.4.1–2.4.3; 2.5.1
Predictors	9b	D;E	Clearly define predictors, how and when measured, and any blinding.	2.1.6; 2.2; 2.3.1; 2.4.1–2.4.3
Predictors	9c	D;E	If predictor measurement required subjective interpretation, describe assessors.	Not applicable
Sample size	10	D;E	Explain how study size was determined and justify adequacy.	2.1.1; 4.7
Missing data	11	D;E	Describe how missing data were handled and reasons for omissions.	2.5.2; Table 1 footnotes
Analytical methods	12a	D	Describe how data were used for model development and performance evaluation.	2.4; 2.5.4; 2.6
Analytical methods	12b	D	Describe how predictors were handled (transformations, standardization, encoding).	2.3.2; 2.5.2; 2.5.4
Analytical methods	12c	D	Specify model types, rationale, model-building steps, tuning, and internal validation.	2.4.1–2.4.3; 2.5.1–2.5.4; 2.6.1
Analytical methods	12d	D;E	Describe heterogeneity handling and clustering considerations.	2.1.4–2.1.5; 2.6.4; 3.4; 4.3–4.5
Analytical methods	12e	D;E	Specify all performance measures and plots used.	2.6.2–2.7.1; 3.5
Analytical methods	12f	E	Describe any model updating (e.g., recalibration) arising from evaluation.	3.5.1; 4.6–4.7
Analytical methods	12g	E	For model evaluation, describe how predictions were calculated.	2.5.4; 2.6.1; 2.8.3
Class imbalance	13	D;E	State why and how class imbalance methods were used.	2.5.3; 3.5.2
Fairness	14	D;E	Describe any approaches used to address model fairness.	Not reported
Model output	15	D	Specify model output and any classification thresholds/rationale.	2.6.2; 3.4–3.5
Development versus evaluation	16	D;E	Identify differences between development and evaluation data.	2.4; 2.6; 3.5.4
Ethical approval	17	D;E	Name ethics board and consent/waiver.	2.1.2; end matter statements

OPEN SCIENCE / PPI

Section/Topic	Item	D/E	Checklist item	Reported in manuscript
Funding	18a	D;E	Give funding source and role of funders.	Funding statement
Conflicts of interest	18b	D;E	Declare conflicts of interest.	Conflicts of Interest statement
Protocol	18c	D;E	State where protocol can be accessed, or that none was prepared.	Protocol Statement

Section/Topic	Item	D/E	Checklist item	Reported in manuscript
Registration	18d	D;E	Provide study registration, or state not registered.	Registration Statement
Data sharing	18e	D;E	Provide details of data availability.	Data Availability Statement
Code sharing	18f	D;E	Provide details of analytical code availability.	2.8.3
Patient & public involvement	19	D;E	Report patient/public involvement or state none.	Patient and Public Involvement Statement

RESULTS

Section/Topic	Item	D/E	Checklist item	Reported in manuscript
Participants	20a	D;E	Describe participant flow and numbers with/without outcome.	Figure 1; 3.1
Participants	20b	D;E	Report characteristics, key predictors, sample size, events, follow-up, and missing data.	Table 1; 3.1
Participants	20c	E	For evaluation, compare predictor/outcome distributions with development data.	Not applicable
Model development	21	D;E	Specify number of participants and events in each analysis.	Figure 1; 3.1; 3.2; Tables 3–5; 3.5
Model specification	22	D	Provide the full model so new predictions can be generated.	2.8.3
Model performance	23a	D;E	Report performance estimates with confidence intervals.	3.4–3.5; Figures 8–9
Model performance	23b	D;E	If examined, report heterogeneity in performance across clusters.	2.6.4; 3.4; 4.3–4.5
Model updating	24	E	Report results from any model updating.	Not applicable

DISCUSSION / USABILITY

Section/Topic	Item	D/E	Checklist item	Reported in manuscript
Interpretation	25	D;E	Give an overall interpretation in context of objectives and previous studies.	4.3–4.7; 5
Limitations	26	D;E	Discuss study limitations and implications for bias, uncertainty, and generalizability.	4.7
Usability	27a	D	Describe how poor-quality or unavailable inputs should be handled in implementation.	2.2.3; 2.3.3; 4.6–4.7
Usability	27b	D	Specify whether user interaction is required and the expertise needed.	2.2–2.3; 4.6
Usability	27c	D;E	Discuss next steps for future research and applicability/generalizability.	4.7; 5

Supplementary Table S1. Per-spectrum FTIR quality control metrics and QC flags. Per-spectrum QC metrics for all spectra, including Amide I SNR, fingerprint spike count, cosine similarity to the cohort median fingerprint spectrum, and fingerprint baseline area fraction, together with QC flag status and the rule(s) triggering the flag (when applicable).

Sample order	Donor_code	DGF	SNR_AmideI	Spike_count	Cosine_fp	Baseline_frac	QC_flag	QC_reason
1	PT2023/000090	no	80,02030965	53	0,982442953	0,296667487	FALSE	
2	PT2023/000094	no	77,11144256	25	0,991096821	0,254345066	FALSE	
3	PT2023/000114	no	80,76087451	34	0,988983289	0,265760565	FALSE	
4	PT2023/000150	no	2,648543805	5	0,918878584	0,017829454	FALSE	
5	PT2023/000185	no	3,256691438	15	0,909698993	0,168859779	FALSE	
6	PT2023/000182	no	4,342837881	12	0,946379071	0,045412903	FALSE	
7	PT2024/000212	yes	20,63727535	36	0,947429364	0,353738755	FALSE	
8	PT2023/000217	no	74,04792133	43	0,981744479	0,30187281	FALSE	
9	PT2023/000284	yes	84,65954428	8	0,997228846	0,205153692	FALSE	
10	PT2023/000286	no	85,9002089	38	0,989102645	0,262108063	FALSE	
11	PT2023/000422	no	4,510845205	12	0,934831135	0,021985346	FALSE	
12	PT2023/000530	no	79,53116996	24	0,99324274	0,252335495	FALSE	
13	PT2023/000685	no	86,03805211	5	0,921414871	0,294683547	FALSE	
14	PT2023/000784	yes	203,7382406	57	0,866508938	0,091444903	FALSE	
15	PT2023/000784	yes	144,4387236	49	0,913305819	0,252998646	FALSE	
16	PT2023/000876	no	74,01826229	5	0,99841661	0,182489985	FALSE	
17	PT2023/000890	yes	59,64285111	14	0,996642395	0,218659002	FALSE	
18	PT2023/000890	no	59,64285111	14	0,996642395	0,218659002	FALSE	
19	PT2023/000907	yes	67,93543718	18	0,995547205	0,236534056	FALSE	
20	PT2023/000907	no	60,46833807	5	0,99792344	0,184091403	FALSE	
21	PT2023/000917	no	86,63074905	13	0,99648825	0,243204262	FALSE	
22	PT2023/000917	no	75,12509711	11	0,998191068	0,20876768	FALSE	
23	PT2023/000911	no	87,96602176	33	0,993459574	0,248653242	FALSE	
24	PT2023/000981	yes	67,370476	12	0,998130859	0,203728721	FALSE	
25	PT2024/000184	yes	68,40510255	8	0,998844206	0,190273585	FALSE	
26	PT2024/000184	yes	62,12240629	7	0,998710011	0,191847234	FALSE	
27	PT2024/000264	no	66,92791199	5	0,998185074	0,167635666	FALSE	
28	PT2024/000266	no	93,19534828	11	0,99791001	0,21286145	FALSE	
29	PT2024/000299	yes	252,4354844	58	0,841125644	0,02577668	TRUE	low cosine (<0.85)
30	PT2024/000383	no	99,10921811	45	0,32103929	25,54477739	TRUE	low cosine (<0.85); high baseline fraction (>0.5)
31	PT2024/000404	yes	168,8776542	50	0,886846244	0,112372786	FALSE	

32	PT2024/000420	no	57,9385729	6	0,999263626	0,206118173	FALSE	
33	PT2024/000468	no	167,9123188	53	0,887917447	0,148322491	FALSE	
34	PT2024/000488	no	74,17303378	6	0,998754707	0,205349818	FALSE	
35	PT2024/000499	no	250,9422654	51	0,896176496	0,173179594	FALSE	
36	PT2024/000531	no	80,05036934	17	0,994347413	0,248871121	FALSE	
37	PT2024/000615	no	84,29063593	16	0,995370182	0,245502286	FALSE	
38	PT2024/000641	no	75,28603662	9	0,998451139	0,228838561	FALSE	
39	PT2024/000660	no	66,78459685	6	0,998518352	0,171563349	FALSE	
40	PT2024/000677	yes	76,78640518	4	0,995987704	0,138917846	FALSE	
41	PT2024/000778	yes	61,91467225	0	0,99735453	0,158714382	FALSE	
42	PT2024/000861	no	69,81479288	5	0,997482937	0,153029538	FALSE	
43	PT2024/000897	no	41,49260885	0	0,997207911	0,136331573	FALSE	
44	PT2024/000902	no	61,88132196	11	0,995349344	0,137217887	FALSE	
45	PT2024/000918	no	63,17748007	8	0,996183602	0,14772147	FALSE	
46	PT2024/000922	yes	4,13409317	26	0,761865521	0,768242519	TRUE	low cosine (<0.85); high baseline fraction (>0.5)
47	PT2024/000958	no	43,12706818	5	0,99438991	0,104090907	FALSE	
48	PT2024/000958	no	52,69971904	10	0,991189682	0,058274074	FALSE	
49	PT2024/000962	no	57,29168619	0	0,994851339	0,131392631	FALSE	
50	PT2024/000962	no	68,25867993	0	0,997389269	0,160734346	FALSE	
51	PT2025/000040	no	82,5291529	16	0,993801473	0,254959709	FALSE	
52	PT2025/000039	no	285,9335329	58	0,889716302	0,191992811	FALSE	
53	PT2025/000110	no	147,6171959	53	0,902129679	0,23240416	FALSE	
54	PT2025/000277	no	66,88974278	11	0,998815549	0,209408402	FALSE	
55	PT2025/000498	no	68,86725665	27	0,985384323	0,297835621	FALSE	
56	PT2023/000782	no	25,37059904	0	0,779649138	2,540215381	TRUE	low cosine (<0.85); high baseline fraction (>0.5)

QC, quality control; SNR, signal-to-noise ratio; DGF, delayed graft function.

Supplementary Table S2. Exploratory benchmark performance of clinical-only models under standard stratified cross-validation. The prespecified primary internal validation is the donor-blinded grouped analysis reported in Section 3.5. Results are shown for the full cohort (DBD + DCD) and for donor-type data (DCD-only and DBD-only), comparing models trained on all clinical variables with models trained on FCBF-selected predictors.

Dataset evaluated	Feature set	CV	Model	Target class	AUC	Accuracy	Sensitivity	Specificity
All (DBD + DCD)	All variables	Stratified 5-fold	Tree	Average	0.779	0.768	0.768	0.542
				No	0.775	0.768	0.881	0.429

				Yes	0.775	0.768	0.429	0.881
				Average	0.776	0.768	0.768	0.732
			Naive Bayes	No	0.814	0.768	0.786	0.714
				Yes	0.814	0.768	0.714	0.786
				Average	0.855	0.857	0.857	0.667
			Tree	No	0.839	0.857	0.952	0.571
				Yes	0.839	0.857	0.571	0.952
	FCBF-selected			Average	0.834	0.875	0.875	0.768
			Naive Bayes	No	0.869	0.875	0.929	0.714
				Yes	0.863	0.875	0.714	0.929
				Average	0.300	0.538	0.538	0.162
			Tree	No	0.300	0.538	0.000	0.700
				Yes	0.300	0.538	0.700	0.000
	All variables			Average	0.767	0.385	0.385	0.582
			Naive Bayes	No	0.767	0.385	0.667	0.300
				Yes	0.767	0.385	0.300	0.667
DCD only (n=14)				Average	0.717	0.769	0.769	0.464
			Tree	No	0.681	0.769	0.900	0.333
				Yes	0.681	0.769	0.333	0.900
	FCBF-selected			Average	0.800	0.692	0.692	0.908
			Naive Bayes	No	0.778	0.692	0.600	1.000
				Yes	0.833	0.692	1.000	0.600
				Average	0.567	0.907	0.907	0.093
			Tree	No	0.603	0.907	1.000	0.000
				Yes	0.603	0.907	0.000	1.000
	All variables	Stratified 3-fold		Average	0.391	0.140	0.140	0.912
			Naive Bayes	No	0.410	0.140	0.051	1.000
				Yes	0.410	0.140	1.000	0.051
DBD only (n=42)				Average	0.699	0.884	0.884	0.539
			Tree	No	0.628	0.884	0.923	0.500
				Yes	0.628	0.884	0.500	0.923
	FCBF-selected			Average	0.901	0.721	0.721	0.747
			Naive Bayes	No	0.910	0.721	0.718	0.750
				Yes	0.910	0.721	0.750	0.718

DGF, delayed graft function; DBD, donation after brain death; DCD, donation after circulatory death; FCBF, fast correlation-based filter; CV, Cross validation; AUC, area under the receiver operating characteristic curve.

Supplementary Table S3. FCBF feature ranking and selected predictor subsets for clinical benchmarking

Cohort	N_predictors_ranked	N_selected (FCBF_score>0)	Features
All (DBD+DCD)	25	5	Donor Type, Recipient ethnicity, Donor Cause of death, Etiology of kidney disease, Donor serum creatinine
DCD only	25	5	Kidney Donor Risk Index, Recipient sex, Kidney Donor Profile Index, Renal replacement therapy (days), Recipient age at transplant
DBD only	25	4	Renal replacement therapy (days), Recipient age at transplant, Donor serum creatinine, Donor cardiorespiratory arrest

DBD, donation after brain death; DCD, donation after circulatory death

Supplementary Table S4. Exploratory benchmark performance of FTIR-only models for delayed graft function prediction under standard stratified 2-fold cross-validation. The prespecified primary internal validation is the donor-blinded grouped analysis reported in Section 3.5. Results are shown for the full cohort and for donor-type × preservation-solution strata. AUC is reported as NA when a test fold contained only one outcome class; such estimates should be interpreted as non-evaluable or exploratory.

Model	Donor type	Perfusion solution	Feature set	CV	Target class	AUC	Accuracy	Sensitivity	Specificity
Naive Bayes	All	All	All wavenumbers	Stratified 2-fold Cross validation	Average (None)	0.443	0.304	0.304	0.577
					DGF=yes	0.437	0.304	0.714	0.167
					DGF=no	0.457	0.304	0.167	0.714
			FCBF-selected wavenumbers		Average (None)	0.849	0.804	0.804	0.839
					DGF=yes	0.859	0.804	0.857	0.786
					DGF=no	0.866	0.804	0.786	0.857
	DBD	Celsior®	All wavenumbers		Average (None)	0.500	0.081	0.081	0.919
					DGF=yes	0.500	0.081	1.000	0.000
					DGF=no	0.338	0.081	0.000	1.000
			FCBF-selected wavenumbers		Average (None)	0.946	0.216	0.216	0.931
					DGF=yes	0.978	0.216	1.000	0.147

			DGF=no	0.971	0.216	0.147	1.000
	Custodiol®/HTK	All wavenumbers	Average (None)	0.200	0.333	0.333	0.067
			DGF=yes	NA	0.333	0.000	0.400
			DGF=no	NA	0.333	0.400	0.000
		FCBF-selected wavenumbers	Average (None)	0.200	0.833	0.833	0.167
			DGF=yes	NA	0.833	0.000	1.000
			DGF=no	NA	0.833	1.000	0.000
DCD	Celsior®	All wavenumbers	Average (None)	0.389	0.182	0.182	0.818
			DGF=yes	0.300	0.182	0.000	1.000
			DGF=no	0.500	0.182	1.000	0.000
		FCBF-selected wavenumbers	Average (None)	0.861	0.545	0.545	0.899
			DGF=yes	0.950	0.545	0.444	1.000
			DGF=no	0.950	0.545	1.000	0.444
	Custodiol®/HTK	All wavenumbers	Average (None)	0.000	0.000	0.000	0.000
		FCBF-selected wavenumbers	Average (None)	0.000	0.000	0.000	0.000

DBD, donation after brain death; DCD, donation after circulatory death FCBF, fast correlation-based filter; CV, Cross validation; AUC, area under the receiver operating characteristic curve.

Supplementary Table S5. Exploratory benchmark performance of combined clinical + FTIR models under standard stratified cross-validation. The prespecified primary internal validation is the donor-blinded grouped analysis reported in Section 3.5. Results are shown for the full cohort and for donor-type strata, using either the full combined feature set or an FCBF-selected subset; subgroup estimates should be interpreted as exploratory because of sparse events.

Model	Donor type	Feature set	CV	AUC	Accuracy	Sensitivity	Specificity
Naïve Bayes	All (DBD + DCD)	All features	Stratified 3-fold Cross validation	0.782	0.873	0.873	0.768
	All (DBD + DCD)	FCBF-selected		0.887	0.891	0.891	0.822
	DBD only	All features		0.500	0.095	0.095	0.905
	DBD only	FCBF-selected		0.980	0.952	0.952	0.995
	DCD only	All features		0.733	0.692	0.692	0.908

DBD, donation after brain death; DCD, donation after circulatory death FCBF, fast correlation-based filter; CV, Cross validation; AUC, area under the receiver operating characteristic curve.
